# Exergy Analysis of Sulfuric Acid Production: A Systematic Framework Using UniSim Design

**DOI:** 10.1021/acsomega.5c12233

**Published:** 2026-02-27

**Authors:** Ulysses Guilherme Ferreira, Thiago Vaz da Costa, Sérgio Mauro da Silva Neiro

**Affiliations:** School of Chemical Engineering, 28119 Uberlândia Federal University (UFU), Av João Naves de Ávila, 2121, bloco 5K, sala 225, Santa Mônica, Uberlândia, MG 38408-100, Brazil

## Abstract

This study proposes a systematic framework for exergy analysis of chemical processes implemented directly in UniSim Design and demonstrates its application to sulfuric acid production via the double contact process. Thermal, mechanical, and chemical exergies are evaluated within the simulator, enabling an integrated assessment of unit- and plant-level irreversibilities. Results indicate that the sulfur-burning furnace and major heat exchangers account for more than 70% of total exergy destruction. Increasing steam pressure from 42 to 60 bar reduces lost work by 2381 kW (1.33%) and increases potential power generation by 16%, whereas optimization of selected heat-exchanger outlet temperatures yields only marginal improvements. Process rearrangement of the furnace and heat recovery section confirms that the associated irreversibilities are unavoidable and endogenous. The overall plant exergy efficiency is 21.02%, increasing to 48.25% when product stream exergy is included. Grassmann diagrams are used to visualize exergy flows and losses. The results demonstrate UniSim Design as a robust and transparent platform for applied exergy analysis of industrial processes.

## Graphical abstract

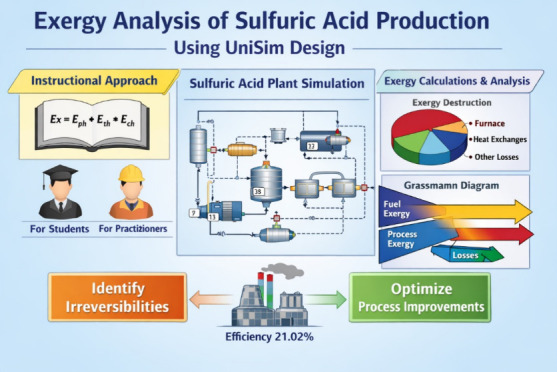

## 1 Introduction

The chemical industry stands out as one of the most energy-intensive sectors globally, accounting for approximately 30% of total industrial energy consumption.^1^ This significant energy demand, coupled with rising expenses related to raw materials, infrastructure, and utilities, continues to place substantial pressure on operating margins and profitability.^2–4^ In response, improving energy efficiency has become a strategic priority across industrial sites. Implementing best practices in process design and operation is not only essential for reducing energy consumption and operating costs, but also for advancing broader goals of sustainability and emissions reduction.

Among the various methodologies developed to support energy optimization, exergy analysis has emerged as a particularly powerful thermodynamic tool. Unlike conventional energy balances, exergy analysis accounts for both the quantity and quality of energy transformations within a system. By evaluating irreversibilities and identifying where useful work is lost, exergy-based methods enable a deeper understanding of process inefficiencies. This makes exergy analysis especially valuable for diagnosing underperforming components and prioritizing improvement efforts in thermal and chemical systems.

Although exergy analysis has long been explored in academic research, its practical implementation in industrial contexts has gained increasing momentum in recent years,^5,6^ particularly as commercial process simulation tools become more accessible and user-friendly. Borreguero et al.^7^ demonstrated the educational value of process simulation by showing that simulator-based learning using Aspen Plus and Aspen HYSYS significantly enhances students’ problem-solving skills, industrial preparedness, and conceptual understanding. While pedagogical in scope, this work highlights the broader role of simulation platforms in facilitating the dissemination and adoption of advanced thermodynamic and exergy-based methodologies.

Recent efforts have investigated the use of commercial process simulation environments, such as UniSim Design,^8,9^ Aspen HYSYS,^10,11^ and Aspen Plus^12,13^ as platforms to support exergy analysis. While these platforms offer high-fidelity modeling of complex chemical processes, rigorous thermophysical property databases, and automated mass and energy balances, exergy analysis is still not natively embedded within their core functionalities. In practice, exergy evaluations, particularly chemical exergy calculations, are typically performed through external spreadsheets, postprocessing scripts, or user-developed routines. This fragmented workflow limits automation, scalability, and reproducibility, and constrains the systematic use of exergy-based methods for design, optimization, and decision-making. As a result, despite the advanced simulation capabilities of commercial tools, there remains a clear gap in the development of fully integrated exergy analysis frameworks that seamlessly couple rigorous process simulation with comprehensive exergy accounting.

Despite its growing relevance, exergy analysis remains underutilized in chemical engineering analysis, in part due to a lack of clear instructional resources that demonstrate how to carry out such analyses using commercial simulation tools. Many practitioners and students are unfamiliar with how to formulate exergy balances, obtain necessary data from simulations, and interpret the results in a meaningful way. Existing literature often presents exergy results without detailing the methodological steps required to replicate the analysis, which can discourage wider adoption and limit the method’s practical impact.

This manuscript aims to address that gap by providing a step-by-step guide for conducting exergy analysis using UniSim Design, demonstrated through an industrially significant case study: a sulfuric acid production plant. Intended for chemical engineering students and professionals, the manuscript begins with a concise overview of the thermodynamic foundations of exergy, including the distinction between physical, chemical, and total exergies. It then introduces the sulfuric acid process and the model developed in UniSim Design and walks the reader through the key stages of system definition, data extraction, and exergy calculation. Special emphasis is placed on practical implementation, how to obtain enthalpies, entropies, and flow properties from simulation outputs and use them to construct exergy balances for major process units. By anchoring the methodology in a realistic and industrially relevant system, this work not only reinforces theoretical understanding but also equips readers with the skills and tools needed to apply exergy analysis in real-world chemical engineering contexts.

In this manuscript, we also aim to present clear definitions and a consolidated nomenclature for the various exergy-related analyses, providing a consistent reference for technical terms commonly used in the field. This serves as a practical guide for readers to become familiarized with the terminology and concepts, facilitating a better understanding and application of exergy-based methodologies in future studies.

By combining thermodynamic theory with simulation practice, this framework empowers engineers to apply exergy concepts in real-world contexts. The approach not only builds technical competence in process analysis but also fosters a mindset of critical evaluation and continuous improvement, key attributes for designing more energy-efficient, cost-effective, and environmentally sustainable chemical processes.

The manuscript is organized as follows: Section 2 presents the literature review, highlighting recent contributions and research gaps in exergy-based assessments. Section 3 introduces the fundamental concepts and formulations of exergy analysis, while Section 4 applies these principles to the simulated process, presenting the results and discussion. Finally, Section 6 concludes with the main findings and outlines perspectives for future developments.

## 2 Literature Review

The growing demand for energy-efficient, low-emission, and economically viable process systems has motivated the widespread adoption of exergy-based analysis methods as advanced diagnostic and decision-support tools. Unlike conventional energy analysis, exergy analysis enables the identification of irreversibilities and quality losses associated with real processes, thereby providing deeper insight into system inefficiencies. Over the past three decades, this framework has been progressively extended through advanced exergy, exergoeconomic, and exergoenvironmental analyses, allowing inefficiencies to be decomposed into avoidable/unavoidable and endogenous/exogenous contributions, and explicitly linking thermodynamic losses to economic costs and environmental impacts.

Advanced exergy analysis was first comprehensively applied to separation systems by Wei et al.,^14^ who introduced an exergoeconomic framework for distillation based on the decomposition of exergy destruction and investment costs into avoidable and unavoidable parts. By defining reference conditions linked to theoretical performance limits, they demonstrated that conventional exergoeconomic indicators may misguide improvement priorities when avoidability is ignored. Application to a light-ends separation plant revealed substantial avoidable inefficiencies in selected columns and utility heat exchangers, motivating a modified exergoeconomic factor better suited for cost-effective decision-making.

Environmental aspects were formally incorporated into advanced exergy analysis by Manesh et al.^15^ through exergoenvironmental analysis, which links component-level exergy destruction to life-cycle-based environmental indicators. A key contribution was the decomposition of environmental impacts into avoidable and unavoidable components, enabling the identification of realistically mitigable environmental burdens. This framework was later combined with advanced exergetic and exergoeconomic analyses for the optimal design of a cogeneration system in the Iran LNG plant, where enhanced graphical tools revealed thermodynamic, economic, and environmental trade-offs more clearly than conventional R-curve analysis.

Advanced exergy-based methods have been extensively applied to power generation systems. Petrakopoulou et al.^16^ applied advanced exergoeconomic analysis to a combined-cycle power plant and showed that the combustion chamber dominates exergy destruction, largely unavoidable and endogenous, while turbines and heat exchangers exhibit non-negligible avoidable costs. Subsequent studies evaluated low-emission technologies, including chemical looping combustion,^17^ hydrogen-fueled combined cycles with precombustion capture,^18^ and postcombustion capture using monoethanolamine,^19,20^ consistently revealing significant efficiency penalties and increased electricity costs, but also identifying improvement potential in auxiliary and compression subsystems.

Near-zero-emission power generation systems have been widely analyzed using advanced exergy-based methods. Studies on oxy-fuel combustion and hybrid solid oxide fuel cell-combined cycle plants showed that additional irreversibilities introduced by carbon capture can be partially offset by higher overall power generation.^21,22^ Chemical looping combustion has been identified as a promising alternative, with advanced exergy analysis revealing that most exergy destruction is unavoidable and endogenous, while the reactor unit and gas turbine components retain meaningful improvement potential.^23^ Life-cycle assessments further indicate that postcombustion capture increases environmental impacts per unit of electricity, whereas chemical looping offers modest advantages, particularly for coal-based systems.^24^ Strong internal interdependencies observed in oxy-fuel plants employing mixed-conducting membranes underscore the need for integrated, system-level optimization rather than isolated component improvements.^25^

Recent studies have emphasized detailed simulation and optimization. Yin et al.^26^ combined computational fluid dynamics and exergy analysis for a large coal-fired boiler, identifying air preheating as a major lever for reducing exergy destruction. Analyses of combined-cycle and gas turbine power plants showed that higher turbine inlet temperatures and pressure ratios improve efficiency, while elevated ambient temperatures significantly degrade performance.^27,28^ Moving beyond diagnosis, Abdulsitar et al.^29^ demonstrated that integrated energy, exergy, and economic optimization can simultaneously enhance efficiency, power output, and economic performance through coordinated adjustment of operating conditions. Khaleel et al.^30^ performed a comparative energy and exergy analysis of coal and gas-fired steam power plants, identifying combustion chambers as the main source of exergy destruction and boilers and steam turbines as the dominant contributors to overall exergy losses. Their study reported an overall exergy efficiency of about 20% and highlighted key opportunities for improving the performance of existing thermal power plants.

Methodological advances aimed at industrial applicability include the computational framework proposed by Gourmelon et al.,^31^ which combines the fuel-product model with transit exergy to enable automated and consistent exergy efficiency calculations in simulation environments. This approach was extended by Gourmelon et al.^32^ through the integration of pinch analysis, energy optimization, and case-based reasoning into a decision-support system demonstrated for an ammonia plant. Valverde et al.^33^ proposed a user-friendly methodology for calculating physical, chemical, and total exergy, as well as exergy destruction and efficiency, by integrating Aspen HYSYS with MS Excel VBA via OLE automation. Validated across seven case studies ranging from unit operations to full process systems, the approach enables real-time exergy evaluation and supports systematic comparison of alternative process configurations.

Advanced exergy analysis has also been applied to heating and district energy systems. Açıkkalp et al.^34^ showed that environmental impacts in building heating systems are predominantly exogenous and unavoidable, with strong sensitivity to ambient temperature, while Yürüsoy and Keçebaş^35^ reported substantially lower environmental impacts for geothermal district heating compared to biomass-based alternatives. In the context of carbon utilization, Huang et al.^36^ compared carbon-to-methanol pathways and found that direct hydrogenation achieves higher exergy efficiency, whereas reverse water–gas shift-based routes yield lower environmental impacts, with reactors and separation units dominating irreversibilities. From a thermoeconomic perspective, De Faria et al.^37,38^ addressed inconsistencies in waste and emission cost allocation by introducing diagram-based frameworks that explicitly account for residue reintegration and environmental pricing.

Recent studies have provided comprehensive reviews of exergy analysis and its applications to emerging energy systems. Sansaniwal et al.^39^ reviewed energy and exergy analyses across a broad range of solar energy applications, demonstrating the versatility of exergy-based assessment beyond fossil-based technologies. Focusing on solar thermal collectors, Murugan et al.^40^ examined the effects of receiver geometry, heat-transfer enhancement techniques, and absorber coatings on energy and exergy efficiencies, highlighting strategies to reduce irreversibilities and thermal losses. Extending this perspective to concentrated solar technologies, Kasaeian et al.^41^ reviewed the exergy performance of solar parabolic dish collectors across diverse applications, identifying receiver and reflector losses as dominant sources of exergy degradation and showing that nanofluids, phase-change materials, and integration with advanced thermodynamic cycles can substantially enhance efficiency. Nanadegani and Sunden^42^ reviewed thermodynamic performance analyses of low and high-temperature fuel-cell systems, emphasizing the role of exergy analysis in complementing conventional energy analysis and the importance of integrating first and second-law approaches with thermoeconomic assessment to capture the effects of operating conditions, fuel selection, and system integration.

Sulfuric acid production is a highly energy-intensive process dominated by strong exothermic reactions and complex heat-integration networks, making it particularly well suited for exergy-based analysis. Accordingly, exergy methods have been increasingly applied to identify irreversibilities, quantify thermodynamic inefficiencies, and guide optimization strategies aimed at improving energy recovery and environmental performance in sulfuric acid plants.

Early exergy-based assessments include the work of Chouaibi et al.,^43^ who analyzed a double-contact, double-absorption sulfuric acid plant and reported an overall exergy efficiency of 55.54%. Their results identified converters and absorbers as the primary sources of irreversibility, highlighting high-temperature reaction and absorption stages as key targets for performance improvement. A foundational thermoeconomic contribution was provided by Almirall,^44^ who developed an integrated exergy and cost-allocation framework for sulfur combustion-based plants using CHEMCAD and a custom thermoeconomic tool.

Beyond steady-state analysis, Kiss et al.^45^ developed a dynamic model of a sulfuric acid plant in gPROMS, demonstrating that sulfur oxide emissions could be reduced by over 40% through operational adjustments without major hardware changes. Although energy recovery was shown to be constrained by thermodynamics, the dynamic framework proved valuable for optimization, control, and operator training. Al-Dallal^46^ analyzed sulfuric acid production via the Wet Sulfuric Acid process using hydrogen sulfide recovered from natural gas, employing Aspen HYSYS simulations to show that feed composition and catalyst volume significantly influence conversion efficiency and that substantial excess heat is available for integration with power generation or heating systems.

High-fidelity simulation approaches have been further advanced in recent years. Mounaam et al.^47^ developed a detailed UniSim Design model of a sulfuric acid plant validated against industrial data, emphasizing its role in digital twin development, operator training, and predictive maintenance. Similarly, Leiva et al.^48^ conducted a simulation-based optimization study of an industrial sulfuric acid plant in Chile using Aspen HYSYS, achieving near-perfect model validation and showing that modest operating adjustments could increase production capacity by 6% while delivering strong economic returns.

More recent research has also focused explicitly on reducing exergy losses and enhancing heat recovery. Wu et al.^49^ and Li et al.^50^ independently proposed heat-recovery improvements using Aspen Plus simulations, showing that additional pumps and heat exchangers can reduce exergy losses by more than 40% and significantly increase steam generation revenue. Galal et al.^51^ analyzed an integrated sulfuric acid plant and steam power station, identifying the waste heat boiler as the dominant source of exergy destruction and demonstrating that improvements in steam conditions and condenser pressure can substantially enhance overall efficiency.

Process optimization has also been addressed through both deterministic and stochastic approaches. Andini et al.^52^ showed using Aspen HYSYS simulations that redesigning heat-exchange configurations and reducing cooling duties improves energy efficiency in contact-process plants, while Mohamed^53^ combined exergy analysis with genetic algorithms to optimize heat-exchanger networks and operating conditions, achieving notable reductions in exergy destruction and increased turbine power output. Agin et al.^54^ introduced a machine-learning-based framework for the catalytic oxidation of sulfur dioxide, combining artificial neural networks with multiobjective optimization to simultaneously improve conversion, productivity, and catalyst cost, signaling a transition toward data-driven and hybrid optimization strategies in sulfuric acid production.

Overall, the reviewed studies demonstrate a clear progression from classical exergy diagnostics toward integrated simulation, optimization, and data-driven approaches in sulfuric acid production. While substantial advances have been made in identifying inefficiencies, enhancing heat recovery, and improving economic performance, most studies remain focused on steady-state analysis and offline optimization. Opportunities remain for integrating advanced exergy analysis with real-time optimization, uncertainty quantification, and digital twin frameworks, particularly as sulfuric acid plants continue to operate under increasingly stringent energy and environmental constraints.

## 3 Exergy Analysis: Concepts and Formulation

Exergy Analysis (EA) provides a framework for assessing the theoretical limits of efficiency in thermodynamic systems, where achieving 100% second-law efficiency would require all process operations to be perfectly reversible, thereby minimizing energy input or maximizing useful output. However, this is impractical and uneconomical, as it would demand infinitely large equipment to eliminate transport gradients. Instead, it is more cost-effective to improve existing processes and design new ones with higher thermodynamic efficiency. Second-law analysis helps identify energy-wasting operations, allowing engineers to focus on areas where energy conservation is most impactful. EA evaluates how much exergy is destroyed due to internal inefficiencies and how much is lost through ineffective handling of waste streams, including both material and energy.^33^

Exergy is a thermodynamic state function derived from the first and second laws of thermodynamics and represents the maximum theoretical useful work (shaft or electrical) that can be extracted as a system moves toward complete equilibrium with its environment, assuming interaction occurs solely between the system and the environment.^55^

According to Ghannadzadeh et al.,^56^ a complete definition of exergy requires an understanding of three fundamental states: the Process State (PS), the Environmental State (ES), and the Standard Dead State (SDS). The PS describes the system’s initial thermodynamic condition, characterized by its temperature, pressure, and composition (*T*, *P*, *z*). The ES represents a restricted equilibrium in which the system is in mechanical and thermal balance with the environment. This means that the system and environment share the same temperature and pressure (*T* ^0^, *P* ^0^, *z*). In contrast, the SDS refers to a condition of full thermodynamic equilibrium, where the system and environment have equal temperature, pressure, and chemical potentials. At this point, no further interactions or state changes can occur, and the system’s exergy is zero (*T* ^0^, *P* ^0^, *z* ^0^). By using *T* ^0^ and *P* ^0^, it is assumed that the environment is at standard conditions. If this is not the case, *T* ^0^ and *P* ^0^ should be replaced throughout by *T* ^00^ and *P* ^00^, which represent the actual (nonstandard) environmental conditions.

As described by Gourmelon et al.,^31^ and neglecting kinetic and potential exergy contributions, the total exergy of a material stream, similar to energy, can be expressed as the sum of its physical and chemical exergy. Physical exergy is the maximum useful work that can be extracted from a system as it moves from its PS to the ES, solely through thermal and mechanical interactions with the environment, without any change in its chemical composition. Chemical exergy is defined as the maximum amount of useful work that can be obtained when a substance is transformed from the ES to the SDS, accounting for differences in chemical composition through ideal chemical reactions with the environment.^56^

The physical exergy component, shown in eq 1, is further divided into thermal exergy, which arises from temperature differences and is given by eq 2, and mechanical exergy, which results from pressure differences and is calculated using eq 3.

$${\mathrm{B̅}}^{\mathrm{Ph}}=\left[\mathrm{H̅}\left(T,P,z\right)-\mathrm{H̅}\left(T^{0},P^{0},z\right)\right]-T^{0}\left[\mathrm{S̅}\left(T,P,z\right)-\mathrm{S̅}\left(T^{0},P^{0},z\right)\right]$$ 1

$${\mathrm{B̅}}^{\mathrm{Th}}=\left[\mathrm{H̅}\left(T,P,z\right)-\mathrm{H̅}\left(T^{0},P,z\right)\right]-T^{0}\left[\mathrm{S̅}\left(T,P,z\right)-\mathrm{S̅}\left(T^{0},P,z\right)\right]$$ 2

$${\mathrm{B̅}}^{\mathrm{Mc}}=\left[\mathrm{H̅}\left(T^{0},P,z\right)-\mathrm{H̅}\left(T^{0},P^{0},z\right)\right]-T^{0}\left[\mathrm{S̅}\left(T^{0},P,z\right)-\mathrm{S̅}\left(T^{0},P^{0},z\right)\right]$$ 3

Here, *H̅* and *S̅* denote the molar enthalpy and molar entropy, respectively; *B̅* ^Ph^, *B̅* ^Th^, and *B̅* ^Mc^ represent the molar physical, thermal, and mechanical exergies. The variables *T*, *P* and *z* refer to the temperature, pressure and composition of the system, while *T* ^0^ and *P* ^0^ denote the temperature and pressure of the environment with which the system is assumed to reach equilibrium and, as stated previously, is assumed to be in standard conditions. In these equations, enthalpy and entropy are combined in a form resembling the Gibbs free energy. However, the entropy term is multiplied by the environment temperature, *T* ^0^, rather than the system’s stream temperature, *T*.

Figure 1 presents a schematic representation of thermal and mechanical exergy, highlighting their relationship to the maximum obtainable work as the system transitions from the PS to the ES. As shown in the figure, when the system temperature is reduced to the environment temperature through a reversible process (for *T* > *T* ^0^), the maximum thermal work is obtained. Subsequently, in a second reversible step, when the system pressure is reduced to the environment pressure (for *P* > *P* ^0^), additional work is generated. Throughout both steps, heat exchange with the environment may occur as the system approaches thermodynamic equilibrium. In cases where *T* < *T* ^0^ and *P* < *P* ^0^, work must be done on the system. These two reversible steps correspond to the thermal and mechanical components of exergy, respectively, and can be quantified using eqs 2 and 3.

**Figure 1:**
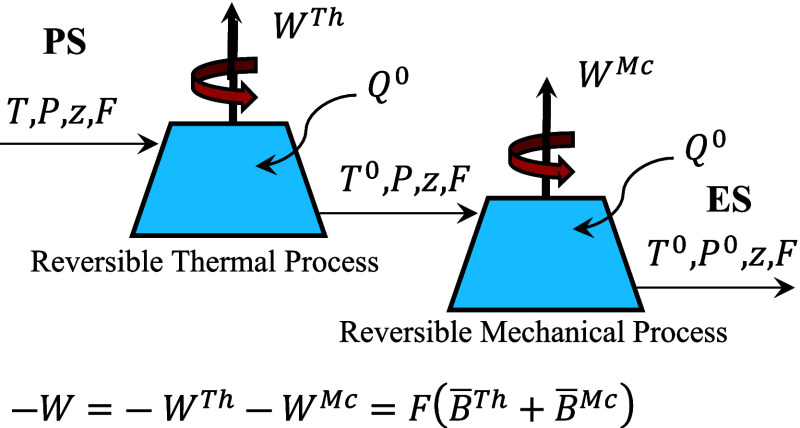
Schematic representation of thermal and mechanical exergy and their relationship to the maximum obtainable work.

Variations in chemical exergy are primarily associated with chemical reactions, mixing or separation of components, and phase changes. According to Kotas,^57^ when the system is brought from ES to the SDS through reversible processes, this transition generally involves both chemical and physical transformations. Chemical processes are required to convert the initial substances within the system into those present in the environment, while the physical processes serve to align the concentrations and physical states of these substances with those of the environment. A substance may undergo a reversible reaction with certain common components of the environment to produce products that are also typical constituents of the environment. For instance, if the substance in question is methane, the corresponding reaction is that shown in eq 4.

$${\mathrm{CH}}_{4}+O_{2}\to {\mathrm{CO}}_{2}+H_{2}O$$ 4

Oxygen participates in the reaction, producing carbon dioxide and water as the final products. These substances are all naturally present in the environment and are also referred to as Reference Substances (RS). The system is designed so that each of them enters and leaves at standard environmental conditions, specifically at pressure *P* ^0^ and temperature *T* ^0^. To determine the chemical exergy of a substance, its work potential is assessed based on the difference in chemical potential between the substance and its environmental counterparts. The RSs represent stable forms of chemical elements in the natural environment, including atmospheric gases, dissolved species in seawater, or solid compounds found on the earth’s surface. If the substance naturally occurs in the environment, only physical transformations are needed to adjust its phase and/or composition.^56^

Based on the preceding discussion, the chemical exergy can be calculated using eq 5 and Figure 2 illustrates the main two steps involved in taking the process stream from the ES to the SDS.

$${\mathrm{B̅}}^{\mathrm{Ch}}\left(T^{0},P^{0},z\right)=\mathrm{H̅}\left(T^{0},P^{0},z\right)-T^{0}\mathrm{S̅}\left(T^{0},P^{0},z\right)-\frac{1}{n}\sum_{i=1}^{N_{c}}\left[\sum_{j=1}^{N_{i}^{\mathrm{ref}}}n_{j,i}\left[{\mathrm{H̅}}_{j}\left(T^{0},P^{0},z^{0}\right)-T^{0}{\mathrm{S̅}}_{j}\left(T^{0},P^{0},z^{0}\right)\right]\right]$$ 5

where *n*_*j*, *i*_ denotes the flow rate of reference substance *j* generated from process substance *i*, and *N*_*i*_^ref^ represents the number of reference substances produced or consumed from process substance *i*. The final form of eq 5 requires identifying whether the process stream mixture exists in the vapor phase, liquid phase, vapor–liquid equilibrium, liquid–liquid equilibrium, or a liquid–liquid–vapor equilibrium. Because the present study considers only pure vapor, pure liquid, or vapor–liquid equilibrium systems, the calculation procedures will be limited to these cases for brevity. For pure vapor, ${\mathrm{B̅}}^{\mathrm{Ch}}\left(T^{0},P^{0},z\right)$ is calculated using eq 6; for pure liquid, eq 7 is applied; and for vapor–liquid mixture, eq 8 is used. For additional details, the reader is referred to Kotas,^57^ Szargut et al.,^58^ Rivero and Garfias,^59^ Ghannadzadeh et al.,^56^ and Gourmelon et al.^31^ where *y*_*i*_ and *x*_*i*_ are the molar fractions of component *i* in the vapor and liquid phases, respectively; ${\mathrm{b̅}}_{i}^{0}$ denotes the standard molar chemical exergy of component *i*; γ_*i*_ is the activity coefficient of component *i*; and ω represents the vapor fraction in the stream mixture.

**Figure 2:**
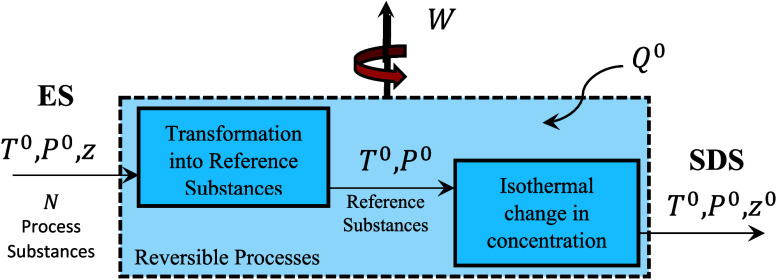
Schematic representation of the steps involved in the calculation of chemical exergy.

$${\mathrm{B̅}}^{\mathrm{Ch}}\left(T^{0},P^{0},z\right)=\sum_{i=1}^{N_{c}}y_{i}\left({\mathrm{b̅}}_{i}^{0}+RT^{0}\ln\left(y_{i}\right)\right)$$ 6

$${\mathrm{B̅}}^{\mathrm{Ch}}\left(T^{0},P^{0},z\right)=\sum_{i=1}^{N_{c}}x_{i}\left({\mathrm{b̅}}_{i}^{0}+RT^{0}\ln\left(x_{i}\gamma_{i}\right)\right)$$ 7

$${\mathrm{B̅}}^{\mathrm{Ch}}\left(T^{0},P^{0},z\right)=\omega \left[\sum_{i=1}^{N_{c}}y_{i}\left({\mathrm{b̅}}_{i}^{0}+RT^{0}\ln\left(y_{i}\right)\right)\right]+\left(1-\omega \right)\left[\sum_{i=1}^{N_{c}}x_{i}\left({\mathrm{b̅}}_{i}^{0}+RT^{0}\ln\left(x_{i}\gamma_{i}\right)\right)\right]$$ 8

The standard molar chemical exergy can be defined for both individual elements and chemical compounds. For a given compound *i*, its standard molar chemical exergy can be estimated based on the standard molar chemical exergy values of its constituent elements ${\mathrm{b̅}}_{j}^{0}$ . In cases where the standard molar chemical exergy of a component is not directly available, it can be determined using reference reactions for which the standard molar chemical exergies of the reactants and products are already tabulated using eq 9.

$${\mathrm{b̅}}_{i}^{0}=\Delta {\mathrm{G̅}}_{f}^{0}+\sum_{j=1}^{N_{i}^{\mathrm{elements}}}n_{i,j}{\mathrm{b̅}}_{j}^{0}$$ 9

When work is involved in a process, its exergy is defined as the amount of useful work that can be extracted from that energy form. As a result, shaft work, whether mechanical or electrical, is fully convertible to useful work and is therefore considered equivalent to exergy, so that eq 10 is valid, where *B* ^*W*^ corresponds to the exergy associated with a work stream.

$$B^{W}=W$$ 10

The exergy of a heat stream is given by eq 11, where *Q* is the heat exchanged and *T̅* is the temperature of the heat source. A heat stream is equivalent to a material stream when its temperature matches the thermodynamic mean temperature *T̅* of the material stream, as defined in eq 12 and illustrated in Figure 3. This temperature assumes reversible heat exchange and is derived from the first and second laws of thermodynamics.

$$B^{Q}=Q\left(1-\frac{T^{{}^{\circ}}}{\mathrm{T̅}}\right)$$ 11

$$\mathrm{T̅}=\frac{{\mathrm{H̅}}^{\mathrm{out}}-{\mathrm{H̅}}^{\mathrm{in}}}{{\mathrm{S̅}}^{\mathrm{out}}-{\mathrm{S̅}}^{\mathrm{in}}}$$ 12

**Figure 3:**
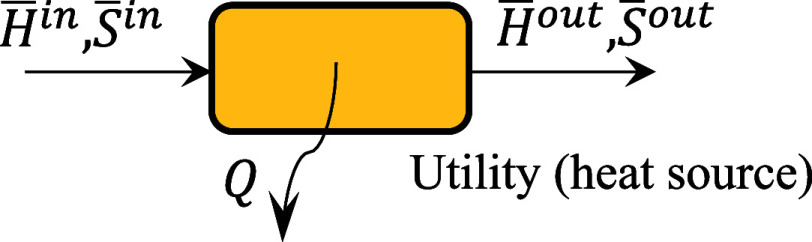
Determination of the thermodynamic mean temperature.

The exergy balance of a system can be written as in eq 13. In real-world applications, no process is perfectly efficient; the exergy supplied to a system invariably surpasses the exergy recovered. This discrepancy arises from irreversibilities caused by the inherent thermodynamic limitations of process operations and wastes. Figure 4 presents a schematic representation of the exergy balance for a system, highlighting the two key factors that prevent it from delivering the maximum possible useful work.

$$\sum_{i=1}^{NS_{mat}^{\mathrm{in}}}B_{mat,i}^{\mathrm{in}}+\sum_{i=1}^{NS_{heat}^{\mathrm{in}}}B_{heat,i}^{\mathrm{in}}+\sum_{i=1}^{NS_{work}^{\mathrm{in}}}B_{work,i}^{\mathrm{in}}=\sum_{i=1}^{NS_{\mathrm{mat}}^{\mathrm{out}}}B_{\mathrm{mat},i}^{\mathrm{out}}+\sum_{i=1}^{NS_{\mathrm{heat}}^{\mathrm{out}}}B_{\mathrm{heat},i}^{\mathrm{out}}+\sum_{i=1}^{NS_{\mathrm{work}}^{\mathrm{out}}}B_{\mathrm{work},i}^{\mathrm{out}}+\sum_{i=1}^{NS_{\mathrm{mat},\mathrm{waste}}^{\mathrm{out}}}B_{\mathrm{mat},\mathrm{waste},i}^{\mathrm{out}}+LW$$ 13

where multiple exergy inputs include material (*NS*_mat_^in^), heat (*NS*_heat_^in^), and work (*NS*_work_^in^), and multiple exergy outputs include material (*NS*_mat_^out^), heat (*NS*_heat_^out^), and work (*NS*_work_^out^). The term *LW* denotes irreversibilities, commonly referred to as lost work, which arise from entropy generation and must therefore always be positive. The summation over material waste (*NS*_mat, waste_^out^) includes all material streams discharged to the environment without recovering their useful work.

**Figure 4:**
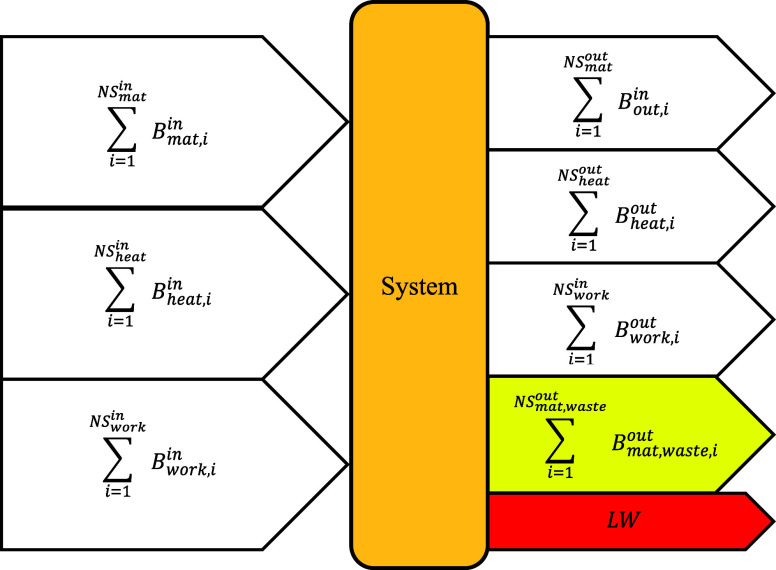
Schematic representation of the exergy balance for a system.

Lastly, conducting a meaningful exergy analysis requires the establishment of performance indicators that assess the exergetic efficiency of a process and pinpoint the unit operations where improvements are most needed. Simple exergy efficiency is defined as the ratio of total exergy output to input and, while easy to calculate, it can be misleading because it ignores whether part of the output is actually wasted. Rational efficiency overcomes this by relating the desired exergetic effect of a unit operation to the exergy truly used, requiring a clear definition of the process objective and thus giving a more realistic measure of performance. Intrinsic efficiency goes further by subtracting the transiting exergy, which is seen as flows that remain unchanged and do not participate in the process, from both inputs and outputs, but despite being conceptually rigorous, it is more complex to apply and still does not explicitly capture external losses

In this work, a modified form of the simple exergy efficiency is employed as the performance metric for each piece of equipment to assess energy efficiency. As expressed in eq 13 and illustrated in Figure 4, material waste corresponds to all process streams discharged to the environment without recovery of their useful work. Since these waste streams can be clearly identified in the sulfuric acid process, they are excluded from the outputs considered in the efficiency numerator, when applied.

## 4 Sulfuric Acid Production by the Double Contact Approach

The double contact process is the most widely adopted industrial method for manufacturing sulfuric acid, primarily due to its high conversion efficiency and reduced emissions. This process is designed to convert sulfur dioxide (SO_2_) into sulfuric acid (H_2_SO_4_) through catalytic oxidation and absorption steps, carried out in two distinct stages, known as the double contact process, to maximize conversion efficiency, as described by King et al.^60^ A general overview of the process is depicted in Figure 5.

**Figure 5:**
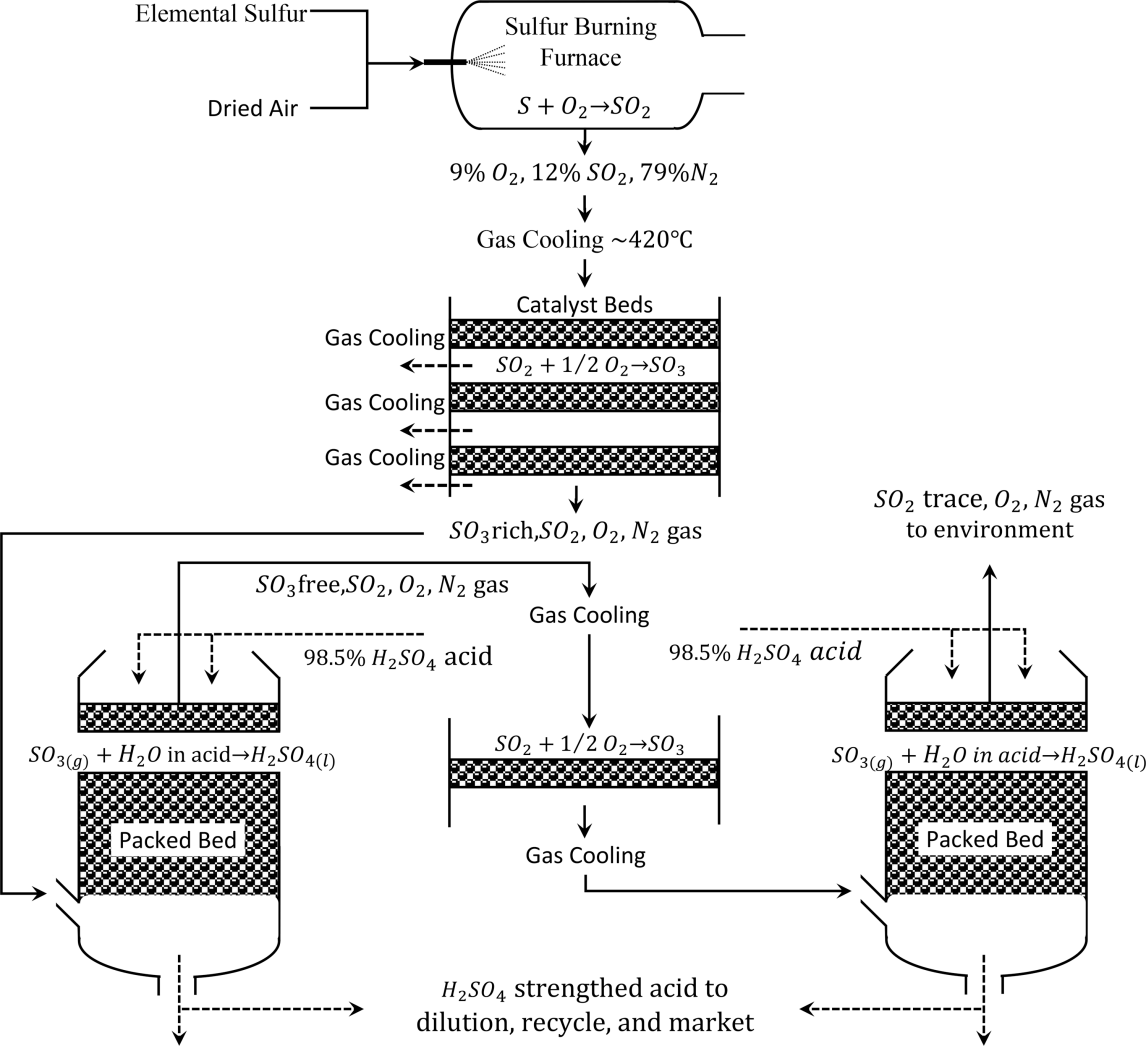
Double contact sulfuric acid manufacture flowsheet.

The process typically begins with elemental sulfur, which accounts for approximately 60% of the global feedstock for sulfuric acid production. The sulfur, often recovered as a byproduct from oil and gas refining, is delivered in molten form or melted on-site using steam coils. It is then atomized and combusted in a specially designed sulfur-burning furnace using dry, filtered air, producing a hot gas mixture containing approximately 12% SO_2_, 9% O_2_, and 79% N_2_ by volume. The combustion is highly exothermic (∼−300 MJ/kmol of sulfur), raising the furnace temperature to around 1150 °C. The furnace off-gas is then cooled to approximately 420 °C in a heat recovery boiler, producing high-pressure steam for electricity generation and process heating.

Before catalytic oxidation, it is essential to remove moisture from the process gas to prevent the formation of corrosive sulfuric acid mist and protect downstream equipment. Drying is achieved by contacting the gas with 98.5% sulfuric acid in a packed drying tower, which chemically absorbs water vapor through an exothermic reaction. This ensures the gas stream is virtually moisture-free (<0.05 g H_2_O/Nm^3^), minimizing corrosion risks.

The dried gas is sent to a catalytic converter, typically composed of four catalyst beds filled with vanadium-based catalysts supported on porous silica. These beds facilitate the exothermic oxidation of SO_2_ to SO_3_ according to the reaction in eq 14.

$${\mathrm{SO}}_{2}+\frac{1}{2}O_{2}\to {\mathrm{SO}}_{3}\left(\Delta H=-100\mathrm{MJ}/\mathrm{kmol}\right)$$ 14

Optimal reaction temperatures range between 400–630 °C. Interstage gas cooling is implemented to control temperature and approach equilibrium conversion limits. After the first pass through the converter, about 85–90% of SO_2_ is converted to SO_3_.

The gas exiting the converter, now rich in SO_3_, enters the intermediate absorption tower, where it is contacted with 98.5% sulfuric acid. SO_3_ reacts with the small amount of water in the acid to form additional H_2_SO_4_ according to eq 15.

$${\mathrm{SO}}_{3}+H_{2}O\left(\mathrm{in}\mathrm{acid}\right)\to H_{2}{\mathrm{SO}}_{4}$$ 15

This step removes most of the SO_3_, resulting in a gas mixture still containing unconverted SO_2_ and excess O_2_. The remaining SO_2_ is then reheated and sent to the second section of the catalytic converter. Here, the same oxidation reaction is performed under carefully controlled conditions to convert most of the residual SO_2_ into SO_3_, further increasing overall conversion efficiency to over 99.5%.

The gas stream undergoes a final absorption step in the final absorber tower, identical in function to the first. SO_3_ is again absorbed into strong sulfuric acid, producing the final product. The tail gas, now with trace SO_2_, is released to the environment through a stack, often after heat recovery and emission monitoring.

The process involves three main sulfuric acid circulation loops: one for drying, and two for absorption. The product acid from both absorption towers is drawn off, cooled, and typically adjusted to market specifications (usually 93–99% H_2_SO_4_) through dilution.

Due to the high exothermicity of sulfur combustion and SO_2_ oxidation, the plant is designed to maximize heat recovery. Heat exchangers and waste heat boilers recover thermal energy to produce steam, which is then used for process heating or converted to electricity via a steam turbine. Some plants operate in cogeneration mode, supplying both steam and power internally or externally.

## 5 Results and Discussion

The reference system for this study is a double contact sulfuric acid production plant with a capacity of approximately 4400 tons per day, described as plant S1 in King et al.^60^ and detailed in the previous section. The process was modeled and simulated using UniSim Design, with additional details on key operations provided in the online Supporting Information. Figure 6 depicts the process flowsheet, in which all process streams are uniquely identified by ascending numerical labels, while the operating conditions of each stream are comprehensively detailed in the stream summary provided in Table S2 in the Supporting Information.

**Figure 6:**
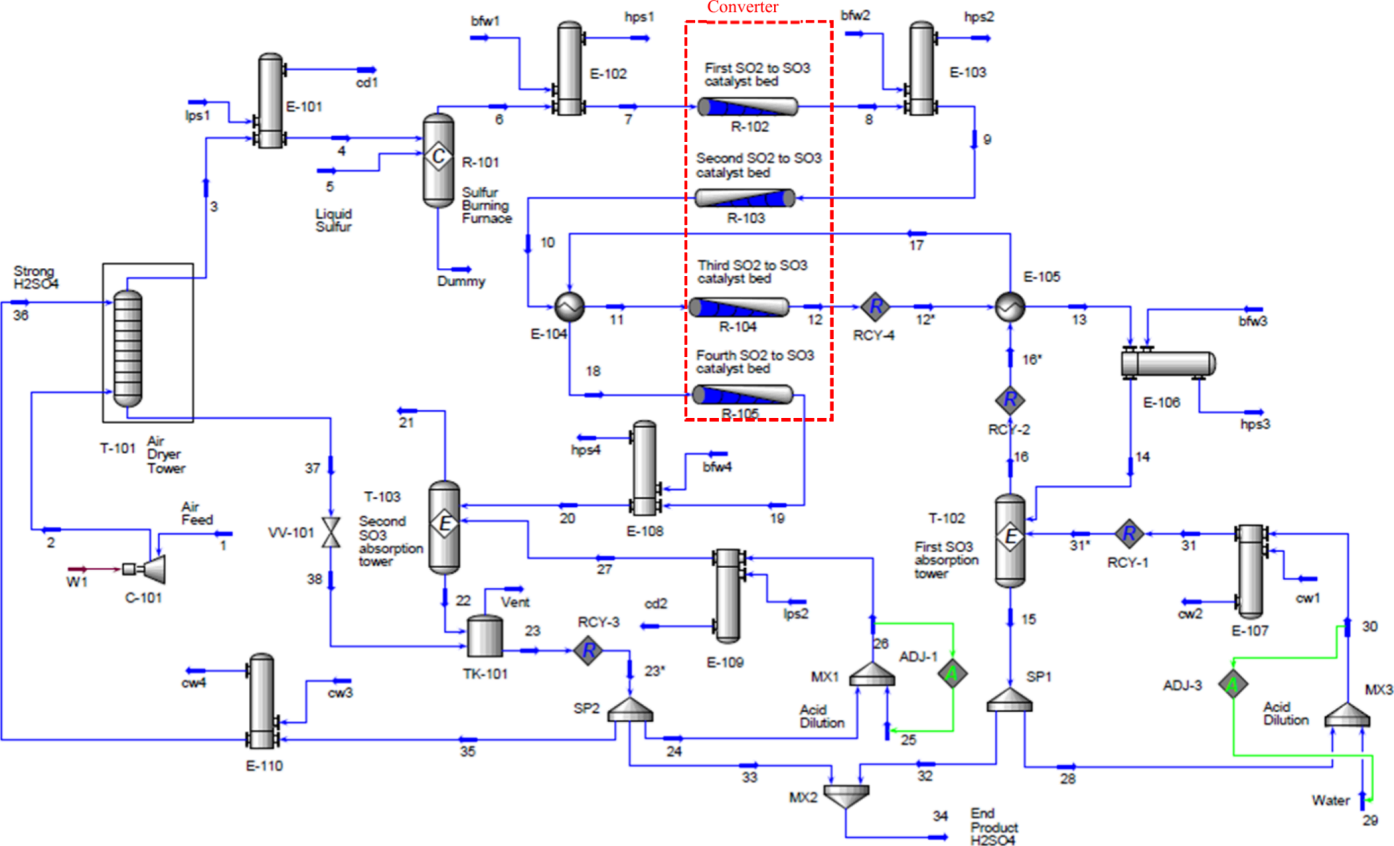
Flowsheet illustrating the sulfuric acid manufacturing process.

The production of sulfuric acid involves highly nonideal liquid solutions, particularly in the absorption and concentration steps where strong molecular interactions between water and sulfuric acid lead to significant deviations from ideal behavior. For this reason, the choice of thermodynamic model is of paramount importance, as an inappropriate model may yield completely erroneous predictions of phase equilibria, enthalpy, and other thermophysical properties, ultimately compromising the reliability of the process simulation. In the present work, the Cubic Plus Association (CPA) thermodynamic package available in UniSim Design is adopted. The CPA model combines a cubic equation of state with an association term to explicitly account for hydrogen bonding and strong polar interactions in mixtures containing water, alcohols, or acids. This hybrid formulation allows it to capture both the volumetric behavior of gases and the highly nonideal liquid phase interactions typical of sulfuric acid systems, providing a robust and reliable basis for property prediction across the wide range of operating conditions encountered in the double contact absorption process. An initial attempt employed the Peng–Robinson equation of state coupled with the NRTL model. However, due to the well-known limitations of NRTL in representing highly nonideal sulfuric acid–water systems, this approach led to calculation inconsistencies, including negative values of lost work, which are thermodynamically impossible.

For the streams representing vapor and cooling water utilities, a separate component list containing only water was created, and the ASME steam thermodynamic model was applied to these streams. Throughout the simulation, pressure drop in heat exchangers were assumed to be negligible on the process side.

Although the literature review reveals numerous contributions applying advanced methodologies to sulfuric acid production, including exergoeconomic and exergoenvironmental assessments, system-level optimization, and energy integration, there remains a gap in studies that present the fundamentals of exergy analysis in a systematic and didactic manner. Most existing works assume prior familiarity with advanced concepts, leaving little guidance for readers or practitioners seeking to build a foundational understanding of how exergy balances are formulated and interpreted in real chemical processes. To address this gap, the present work deliberately focuses on the direct application of basic exergy principles to a simulated sulfuric acid plant. By emphasizing clarity and accessibility rather than optimization or integration, the study provides a pedagogical framework that can serve as a steppingstone for students, engineers, and researchers before engaging with the more complex methodologies that dominate current research. In this context, UniSim Design proves to be a particularly suitable platform, as it couples rigorous thermodynamic models and comprehensive property packages with flexible tools such as integrated spreadsheets and stream-handling functions. These capabilities allow enthalpy, entropy, and chemical exergy values to be computed and exported consistently within a single environment, ensuring both reliability and transparency in the exergy calculations presented here.

The UniSim Design simulation accurately models the double contact process. The simulation workflow mirrors the typical industrial setup and includes major processing blocks for drying, combustion, catalytic conversion, absorption, acid circulation, and heat recovery.

The process begins with the introduction and combustion of liquid sulfur in the sulfur-burning furnace (R-101). The dry air required for combustion is compressed using C-101 and pretreated in an air dryer tower (T-101), where circulating strong sulfuric acid removes moisture. The absorption column used for drying air was configured with five theoretical trays and operated at 140 kPa, assuming no pressure drop. The combustion in the furnace is highly exothermic. As a result, the off-gas exits at 909.3 °C and is primarily composed of SO_2_, O_2_, and N_2_. The hot combustion gases are then cooled in the waste heat boiler (E-102), which recovers thermal energy and generates high-pressure steam (hps1), before the gases enter the first catalytic bed of the converter (R-102). A conversion reactor was selected in UniSim to represent the furnace, with the conversion rate set to 100%.

The cooled gas is directed through R-102 and R-103, representing the first and second catalyst beds of the converter, where SO_2_ is oxidized to SO_3_ over vanadium pentoxide catalysts. In this case, Plug Flow Reactors (PFRs) were used to represent the catalyst beds of the converter (refer to the online Supporting Information for details on the reaction rate and reactor configuration). Pressure drops across each catalyst bed were calculated using the built-in Ergun equation in UniSim Design. The catalyst beds operate under adiabatic conditions. The temperature between the two beds is adjusted to 440 °C using heat exchangers E-103 to maintain optimal conversion conditions. After passing through these two beds, approximately 88% of SO_2_ is converted.

Before entering the third catalyst bed of the converter, the gas exiting the second bed exchanges heat with the gas coming from the first absorption column. Specifically, the gas leaving the top of the first absorption column first exchanges heat with the outlet stream of the third catalyst bed in heat exchanger E-105, increasing its temperature from 116.8 °C to 320.3 °C. It then gains additional heat from the stream entering the third catalyst bed in heat exchanger E104, further raising its temperature to 420 °C. Simultaneously, the temperature of the gas entering the third catalyst bed is adjusted to 445 °C. At the outlet of the third bed, the overall SO_2_ conversion reaches 94%.

The gas mixture, rich in SO_3_, is first precooled in heat exchanger E-105 and then further cooled in heat exchanger E-106 before entering the first SO_3_ absorption tower (T-102). In this simulation, equilibrium reactors were used to model the SO_3_-to-H_2_SO_4_ absorption towers. The gas enters the absorption tower at a temperature of 166 °C, where it comes into contact with 92.8 mol % H_2_SO_4_. In this tower, SO_3_ is converted into H_2_SO_4_ through an exothermic hydration reaction. As a result, the acid stream exiting the tower reaches a composition of 99.99 mol % H_2_SO_4_. The acid within the tower is circulated in a closed loop that includes a stream splitter (SP1) and cooling via heat exchanger E-107 to maintain the desired absorption temperature. The acid is introduced into the absorption tower at 66 °C. A portion of the circulated acid is directed to the product outlet, while water is added within the circulation loop to dilute the acid and maintain its feed composition to the tower at approximately 92.8 mol %.

Unconverted SO_2_ from the first absorption step is reheated in heat exchangers E-104 and E-105, as previously mentioned, and then directed to R-105, which simulates the fourth catalyst bed of the converter. This step increases the overall SO_2_-to-SO_3_ conversion to 99.7%. Cooling between catalyst beds is again managed using heat recovery exchanger E-108. The gas exiting this exchanger, at 135 °C, enters the second absorption tower (T-103), where final SO_3_ absorption takes place. As before, strong sulfuric acid is used, and temperature is controlled through heat exchanger E-109 in a dedicated circulation loop. In this stage, the acid enters the tower at 82 °C, and the exiting acid stream reaches a composition of 99.98 mol %. The final H_2_SO_4_ product is sent to storage tank TK-101. This tank ensures an adequate supply of acid to both the second absorption tower loop (T-103) and the loop serving drying tower for incoming air feed. Acid dilution is again performed in the circulation loop of T-103 to match the composition used in T-102. Before being sent to the drying tower (T-101), the acid is cooled to 45.02 °C using heat exchanger E-110. Another portion of the acid stored in TK-101 is combined with the product from T-102 and directed to the product outlet. The final product achieves a concentration of 99.99 mol %. Tail gases, post final absorption, are released with minimal SO_2_ content (0.00032 mol %). Emissions can be managed through heat exchangers and optional scrubbers.

Table 1 presents the inlet temperatures for each catalyst bed in the converter, together with the SO_2_ conversions and the corresponding outlet temperatures obtained from the simulation. Figure 7 illustrates the conversion as a function of temperature along each catalyst bed. It is worth noting that the equilibrium conversion is reached at the outlet temperature of each bed.

**Table 1:** Inlet and Outlet Temperatures and SO_2_ Conversions for Each Catalyst Bed in the Converter

	bed 1	bed 2	bed 3	bed 4
inlet temperature (°C)	423.0	440.0	445.0	420.0
outlet temperature (°C)	632.0	523.8	465.9	443.7
SO_2_ conversion relative to converter feed	62.76	87.88	94.11	94.28^a^

a Conversion of bed 4 was calculated with respect to its own feed, rather than the overall converter feed.

**Figure 7:**
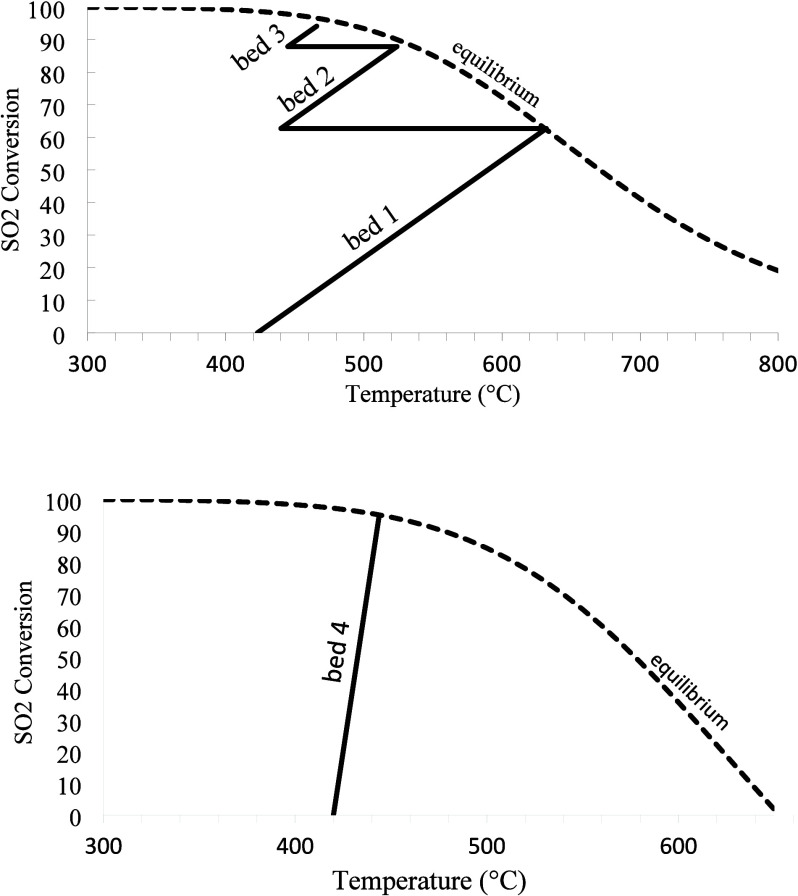
Conversion as a function of temperature along each catalyst bed in the converter.

Utility streams (cooling water: cw1–cw4; boiler feedwater: bfw1–bfw4; high-pressure steam: hps1–hps4) integrate with heat exchangers and boilers to maximize energy recovery, enabling steam generation and waste heat utilization. Their conditions are set according to Turton et al. (2018). The efficiencies of the compressor was set to its default value of 75%.

To carry out the exergy analysis of the sulfuric acid process, the chemical exergy of each process stream is calculated using eqs 6–8. These equations require the standard molar chemical exergy ${\mathrm{b̅}}_{i}^{0}$ , which is typically available as tabulated values for certain components. The ${\mathrm{b̅}}_{i}^{0}$ values adopted in this work were sourced from Kotas^57^ and are reported in Tables 2–4.

**Table 2:** Detailed Calculation of the Chemical Exergy for a Vapor-Phase Process Stream

Stream 8	*y*_*i*_	${\mathrm{b̅}}_{i}^{0}\;\mathrm{kJ}/\mathrm{kmol}$	$y_{i}\left({\mathrm{b̅}}_{i}^{0}+RT^{0}\ln\left(y_{i}\right)\right)\;\mathrm{kJ}/\mathrm{kmol}$
H_2_O	0.01347	11,710	13.8986
H_2_SO_4_	0	161,010	0
N_2_	0.81006	720	160.2388
SO_3_	0.07638	225,070	16,703.3390
O_2_	0.05557	3970	–177.5169
SO_2_	0.04452	303,500	13,169.8768

${\mathrm{B̅}}^{Ch}=\sum {\mathrm{B̅}}_{i}^{Ch}\left(T^{{}^{\circ}},P^{0},z\right)\;\mathrm{kJ}/\mathrm{kmol}$	29,869.8362

**Table 3:** Detailed Calculation of the Chemical Exergy for a Liquid-Phase Process Stream

Stream 34	*x*_*i*_	${\mathrm{b̅}}_{i}^{0}\mathrm{kJ}/\mathrm{kmol}$	$x_{i}\left({\mathrm{b̅}}_{i}^{0}+RT^{0}\ln\left(x_{i}\right)\right)\mathrm{kJ}/\mathrm{kmol}$
H_2_O	5.63 × 10^–5^	11,710	–0.706143
H_2_SO_4_	0.99988	161,010	160,991.7478
N_2_	2.14 × 10^–6^	720	–0.067894
SO_3_	3.89 × 10^–5^	225,070	7.779164
O_2_	8.45 × 10^–7^	3970	–0.025938
SO_2_	1.35 × 10^–5^	303,500	3.7096577
${\mathrm{B̅}}^{\mathrm{Ch}}=\sum {\mathrm{B̅}}_{i}^{\mathrm{Ch}}\left(T^{0},P^{0},z\right)\mathrm{kJ}/\mathrm{kmol}$			161,002.4366

**Table 4:** Detailed Calculation of the Chemical Exergy for a Mixed-Phase Process Stream

Stream 30	*y*_*i*_	*x*_*i*_	${\mathrm{b̅}}_{i}^{0}\mathrm{kJ}/\mathrm{kmol}$	$x_{i}\left({\mathrm{b̅}}_{i}^{0}+RT^{0}\ln\left(x_{i}\right)\right)\mathrm{kJ}/\mathrm{kmol}$	$y_{i}\left({\mathrm{b̅}}_{i}^{0}+RT^{0}\ln\left(y_{i}\right)\right)\mathrm{kJ}/\mathrm{kmol}$
H_2_O	0.999076	0.021106	11,710	45.28759759	11,696.88526
H_2_SO_4_	1.043 × 10^–11^	0.978885	161,010	157558.5578	1.02527 × 10^–6^
N_2_	4.109 × 10^–5^	8.88 × 10^–11^	720	–5.03102 × 10^–06^	–0.999099719
SO_3_	0.000614	7.967 × 10^–6^	225,070	1.561175829	127.0397971
O_2_	1.508 × 10^–5^	2.798 × 10^–10^	3,970	–1.41458 × 10^–05^	–0.355188453
SO_2_	0.000254	4.856 × 10^–7^	303,500	0.129888472	71.79230482
	$\sum {\mathrm{B̅}}_{i}^{\mathrm{Ch}}\left(T^{0},P^{0},z\right)\mathrm{kJ}/\mathrm{kmol}$			157,605.5365	11,894.3631
	ω = 0.0518			157,605.5365(1 – ω)	11,894.3631ω
	${\mathrm{B̅}}^{\mathrm{Ch}}\left(T^{0},P^{0},z\right)\mathrm{kJ}/\mathrm{kmol}$			153,309.28	

The thermal, mechanical, and chemical exergy of the 38 process streams in the sulfuric acid production process depicted in Figure 6 were calculated. Thermal and mechanical exergies are obtained in a straightforward manner using eqs 2 and 3, requiring molar enthalpy and entropy values listed in Table S3 of the Supporting Information. These enthalpy and entropy data were determined in UniSim Design under three conditions: (*T*, *P*, *z*), (*T* ^0^, *P*, *z*), and (*T* ^0^, *P* ^0^, *z*).

To illustrate the chemical exergy calculation, the most complex step, three representative streams were selected: one in the vapor phase, one in the liquid phase, and one in the vapor–liquid phase. The outlet stream of the first catalyst bed in the converter (Stream 8) was selected as the representative vapor stream, the final product stream (Stream 34) as the representative liquid stream, and Stream 30 as the representative vapor–liquid stream. For the vapor stream, chemical exergy was calculated using eq 6, while eq 7 was applied to the liquid stream and eq 8 to the mixed-phase stream. The application of eq 6 is straightforward, requiring only the standard molar chemical exergies and the component compositions. Table 2 shows the detailed calculation for Stream 8.

For liquid-phase streams, the calculation requires not only the standard molar chemical exergies and component compositions but also the activity coefficients. These were determined using UniSim ThermoWorkbench included with the UniSim Design installation. It was observed that only the activity coefficients of H_2_O, SO_2_ and SO_3_ deviated from ideality, with values lower than 1. However, since the concentrations of these components in the liquid phase are very small throughout the process and have negligible impact on the chemical exergy of the stream, all activity coefficients were assumed to follow ideal-solution behavior, i.e., γ_*i*_ = 1. The detailed calculations for process stream 34 are shown in Table 3.

For the mixed-phase stream, the vapor fraction, ω, must also be taken into account. The detailed calculation of the chemical exergy of stream 30 is shown in Table 4.

In practice, the physical and chemical exergy calculations were carried out directly within UniSim Design using a combination of available built-in resources, including spreadsheets and live stream copying. To enable the calculation of physical exergy, a copy of each process stream was maintained in a subflowsheet with temperature and pressure fixed at environmental conditions (25 °C and 1 atm), while compositions and flow rates were dynamically linked to the original streams. This ensured that any modification in the process was automatically reflected in the exergy calculations. The lost work of each unit was also evaluated within UniSim Design, with details presented next. An overview of the implementation is provided in Figure 8. Although the figure is visually dense, its purpose is solely to illustrate that UniSim serves as a powerful and self-contained platform for exergy analysis. Moreover, by automating the exergy calculations, the user can further leverage built-in functionalities such as optimization tools, which could be applied to minimize the total lost work. Thus, UniSim Design offers a robust and comprehensive platform for exergy analysis, consolidating the workflow into a single reliable environment and removing the need for multiple disconnected tools often reported in the literature.

**Figure 8:**
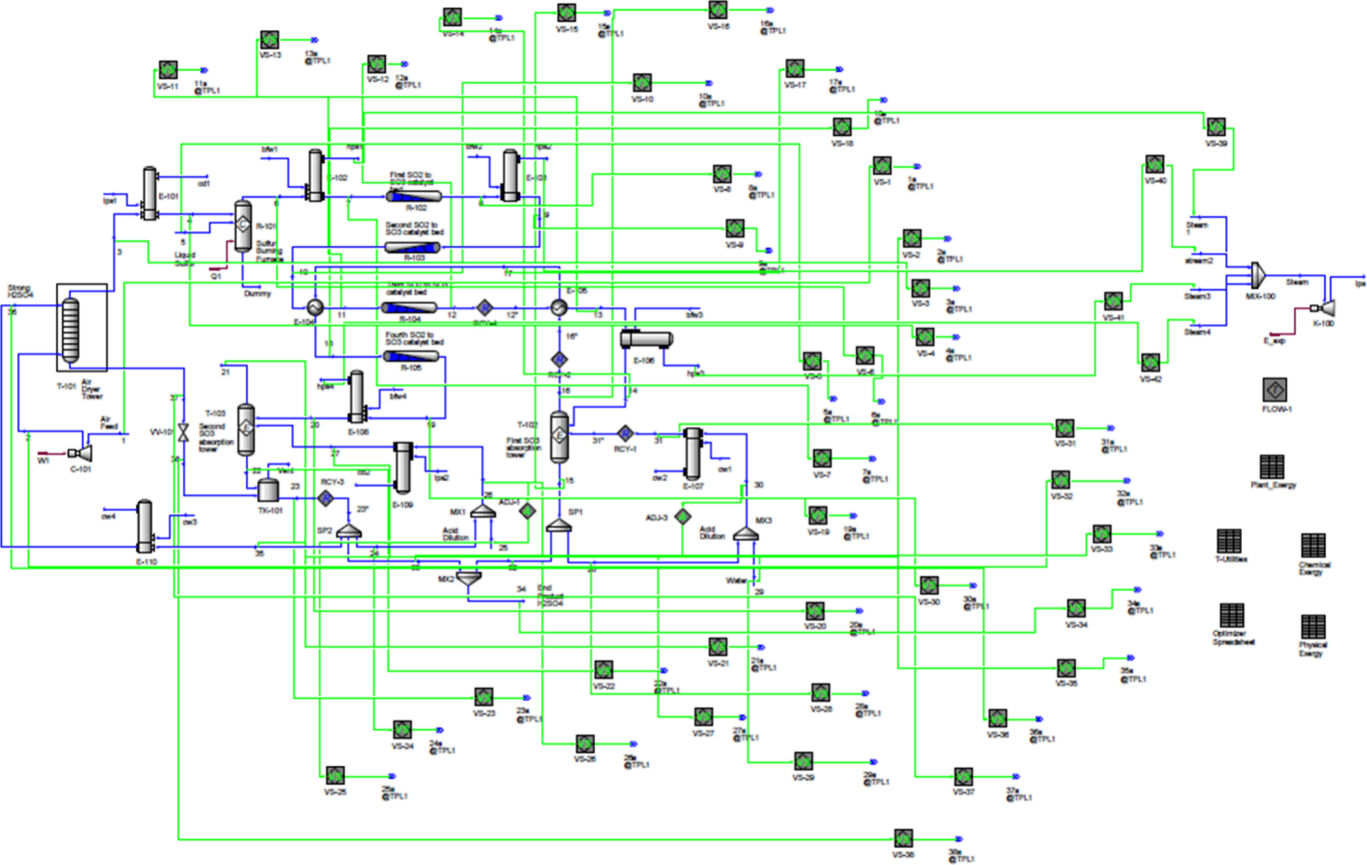
Implementation of physical and chemical exergy calculations in UniSim Design.

The calculated values of the physical exergy components (thermal and mechanical), along with the chemical and total exergies, are provided in Table S4 of the Supporting Information. Using the exergy values of each process stream, the exergy balance expressed in eq 13 and illustrated in Figure 4 was established for every unit within the integrated process flowsheet. The results of these calculations are summarized in Table 5. For conciseness, the exergy associated with heat and work interactions is reported in a single column, where positive values indicate input and negative values indicate output. The last two columns of Table 5 report the lost work and efficiency of each unit, while Figure 9 illustrates the percentage contribution of each unit to the total lost work of the process. The mean temperature *T̅* is calculated based on the inlet and outlet conditions of the utilities in the heat exchangers.

**Table 5:** Summary of Exergy Inputs, Outputs, Lost Work, and Efficiency for Each Process Unit in the Sulfuric Acid Plant

unit	stream in		stream out						
	#	*B*_mat_^in^ (kW)	#	*B*_mat_^out^ (kW)	*T̅*	*B*_heat_ (kW)	*B*_work_ (kW)	*LW* (kW)	%
C-101	1	477.63	2	4354.90			5001.78	1124.50	79.48
E-101	3	4145.46	4	6530.54	432.0	4028.33		1643.25	79.90
E-102	6	231,181.97	7	179,576.77	506.5	–31,391.23		20,213.97	91.26
E-103	8	173,515.12	9	154,818.55	506.5	–12,213.04		6483.54	96.26
E-104	10	152,913.19	11	145,679.18				835.52	99.54
	17	26,790.96	18	33,189.45					
E-105	12	145,232.03	13	132,267.50				3819.89	97.65
	16	17,646.31	17	26,790.96					
E-106	13	132,267.50	14	123,638.27	506.5	–8594.94		34.30	99.97
E-107	30	1,137,936.79	31	1,116,408.51	308.1	–4007.32		17,520.96	98.46
E-108	19	32,661.80	20	15,975.31	506.5	–15004.06		1682.43	94.85
E-109	26	876,254.32	27	881,418.34	432.0	12640.61		7476.59	99.16
E-110	35	1,143,985.11	36	1,140,158.66	308.1	–1414.79		2411.66	99.79
MX1	24	875,870.60	26	876,254.32				4511.34	99.49
	25	4895.05							
MX2	32	78,611.48	34	85,577.93				46.84	99.95
	33	7013.29							
MX3	28	1,138,510.68	30	1,137,936.79				6808.24	99.41
	29	6234.35							
R-101	4	6530.54	6	231,181.97				87,518.97	72.54
	5	312,170.39							
R-102	7	179,576.77	8	173,515.12				6061.65	96.62
R-103	9	154,818.55	10	152,913.19				1905.36	98.77
R-104	11	145,679.18	12	145,232.03				447.14	99.69
R-105	18	33,189.45	19	32,661.80				527.64	98.41
T-101	2	4354.90	3	4145.46				41.18	100.00
	36	1,140,158.66	37	1,140,326.92					
T-102	14	123,638.27	15	1,217,122.16				5278.31	99.57
	31	1,116,408.51	16	17,646.31					
T-103	20	15,975.31	21	8459.81				364.57	99.02
	27	881,418.34	22	888,569.27					
TK-101	22	888,569.27	23	2,026,869.00				2025.57	99.90
	38	1,140,325.29							
VV-101	37	1,140,326.92	38	1,140,325.29				1.63	100.00
total plant								178,785.04	21.02

**Figure 9:**
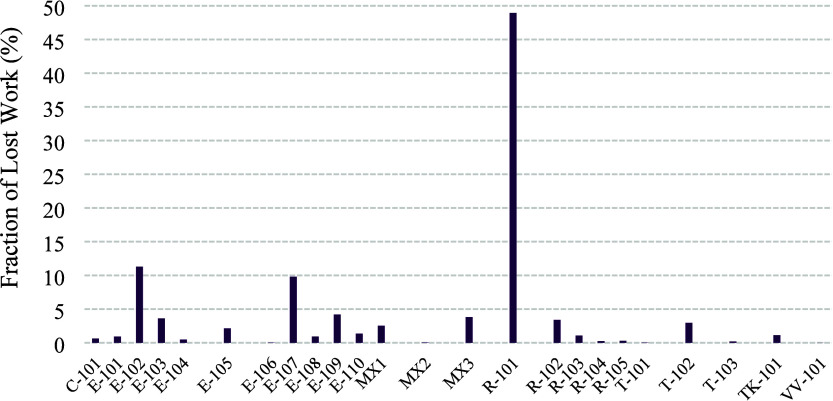
Fraction of lost work for each process unit of the sulfuric acid plant.

In Figure 6, bfw denotes saturated boiler feedwater at 1.7 bar (115.2 °C), lps denotes saturated low-pressure steam at 6 bar (158.8 °C), and hps denotes saturated high-pressure steam at 42 bar (253.2 °C). When vapor is used as the heating medium, only its latent heat is utilized, with condensate leaving the heat exchangers at the same temperature and pressure as the supplied steam. Cooling water (cw) is supplied at 30 °C and exits at 40 °C, always at 1 atm. For completeness and to facilitate visualization of the contribution of each exergy component, the Grassmann diagrams of the main process units are presented in Figures S1–S4 in the Supporting Information.

The exergy analysis highlights that the reactor R-101 is by far the dominant contributor to irreversibilities in the sulfuric acid plant. With a lost work of 87,518.97 kW, this unit alone accounts for nearly half of the total lost work (48.95%). This high value reflects the strongly irreversible chemical transformations occurring in the initial oxidation of sulfur, a step inherently limited by reaction kinetics, heat release, and thermodynamic constraints.

Following R-101, the heat exchanger E-102 is the second most critical contributor, responsible for 20,213.97 kW of lost work (11.31% of the total). This unit handles large thermal gradients associated with high-temperature process gases, making exergy destruction through heat transfer across finite temperature differences a central factor. The third major hotspot is E-107, with 17,520.96 kW of lost work (9.80%). Similar to E-102, this exchanger operates under conditions of substantial exergy degradation due to temperature mismatches.

Together, R-101, E-102, and E-107 alone account for more than 70% of the total lost work in the plant, confirming them as the key targets for efficiency improvement strategies. Secondary contributors (E103, E-105, E-109, R-102, MX3, T-102) while smaller in share, collectively represent another ∼30% of the exergy destruction and should not be overlooked in a comprehensive optimization effort.

In contrast, smaller contributors such as mixers (MX1, MX3) represent mostly unavoidable irreversibilities, as mixing inherently increases entropy. Their improvement potential is limited compared to reactors and exchangers.

A comparison between the efficiency and the percentage contribution to lost work of the process units reveals a clear inverse trend. Units that exhibit higher efficiencies generally contribute less to the overall irreversibilities of the process, while those with lower efficiencies stand out as significant sources of lost work. This relationship emphasizes the role of exergy destruction in shaping unit performance, as equipment with greater dissipation of useful work inevitably limits process efficiency. It is also observed that some units occupy an intermediate position, displaying moderate efficiencies alongside noticeable but not dominant contributions to lost work. Such patterns allow for distinguishing between units that already operate near their thermodynamic potential and those that require closer attention, serving as a guideline for prioritizing optimization efforts.

The overall efficiency of the plant is relatively low, at 21.02%, mainly because the exergy of the outgoing material streams is not considered in the calculation, as this exergy is not recovered or utilized within the process in our simulation. If the exergy of these streams were included, the overall plant efficiency would rise substantially, reaching 48.25%.

The Grassmann diagram shown in Figure 10 provides a clear visualization of the exergy pathways across the key units E-101, R-101, and E-102. In the Grassmann diagram shown in Figure 10, as well as in the subsequent figures, Th denotes thermal exergy, Ph physical exergy, and Ch chemical exergy. In R-101, the most striking feature is the massive inflow of chemical exergy associated with the sulfur feed (stream 5), which reaches approximately 311,736 kW. Nearly half of this exergy is not preserved in chemical form but is instead converted into thermal exergy in the reactor outlet stream (72,771 kW) and into lost work (87,519 kW), highlighting once again the highly irreversible nature of the sulfur oxidation step. This observation aligns with the quantitative results in Table 5, confirming R-101 as the dominant source of irreversibilities in the process.

**Figure 10:**
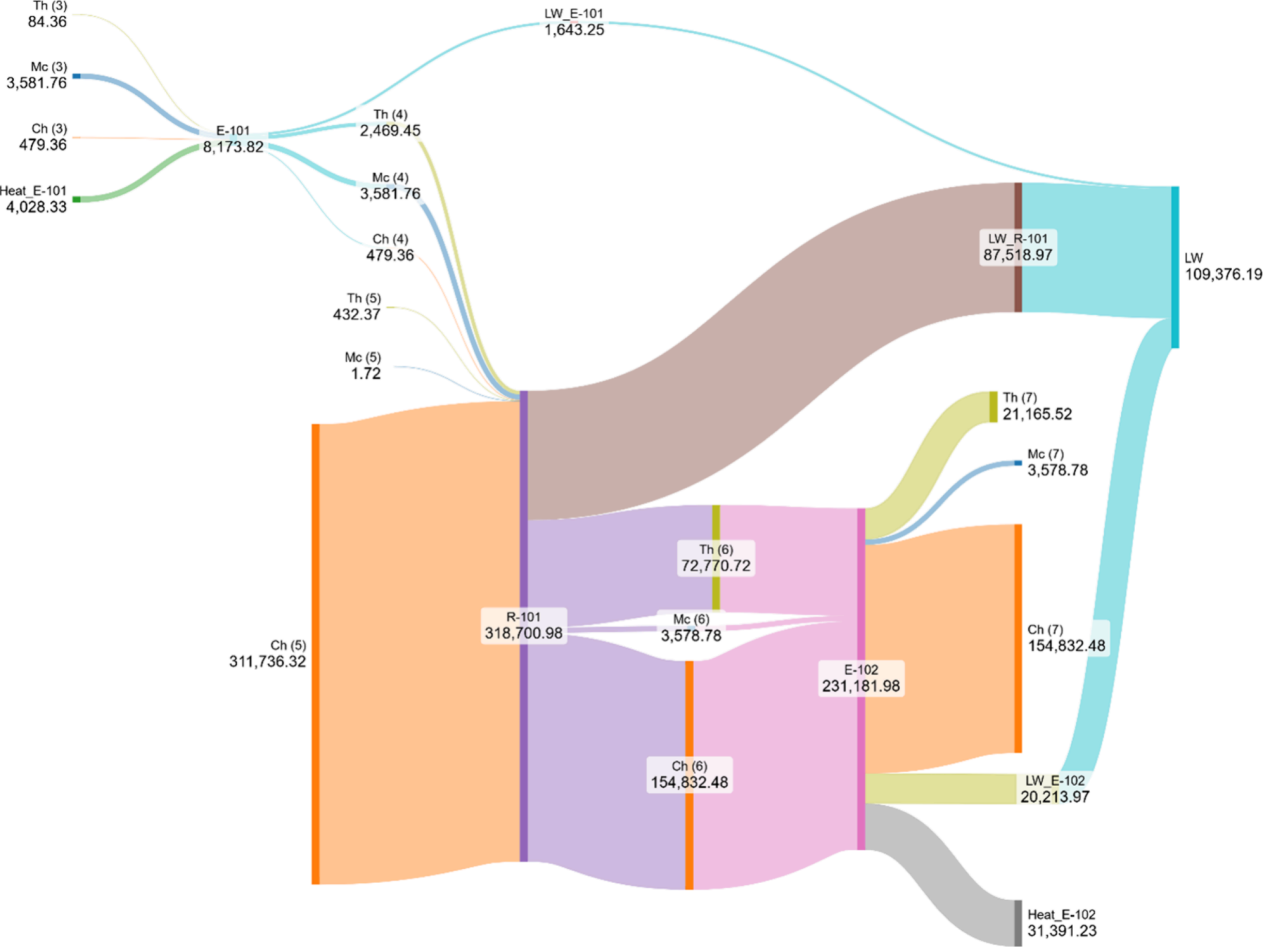
Exergy distribution and lost work in the sulfur burner and associated heat exchangers.

Upstream, E-101 conditions the feed and contributes relatively modest exergy losses (1,643 kW) compared to the reactor. Nevertheless, its role is relevant since it delivers the necessary heat duty to achieve the reaction conditions in R-101, thus coupling thermal and chemical exergy flows.

Downstream, E-102 plays a crucial role in redistributing the large amount of thermal exergy leaving R-101. A significant fraction of this exergy (31,391 kW) is usefully recovered as steam generation, while another substantial portion (20,214 kW) is destroyed as lost work, reflecting the penalty of transferring heat across finite temperature differences. This illustrates the trade-off between energy recovery and exergy destruction that is typical of high-temperature gas cooling in sulfuric acid plants.

Overall, the diagram underscores two central insights: (i) the reactor R-101 dominates irreversibility due to chemical reaction constraints, and (ii) the heat exchanger E-102 is critical for energy integration, converting thermal exergy into valuable steam but still suffering significant destruction. These findings suggest that improving heat recovery strategies could have the greatest impact on reducing global exergy losses in the plant.

The Grassmann diagram for the sequence R-102 to R-104 and their associated heat exchangers (E-103 and E-104) shown in Figure 11 illustrates the progressive transformation of exergy along the catalytic section of the process.

**Figure 11:**
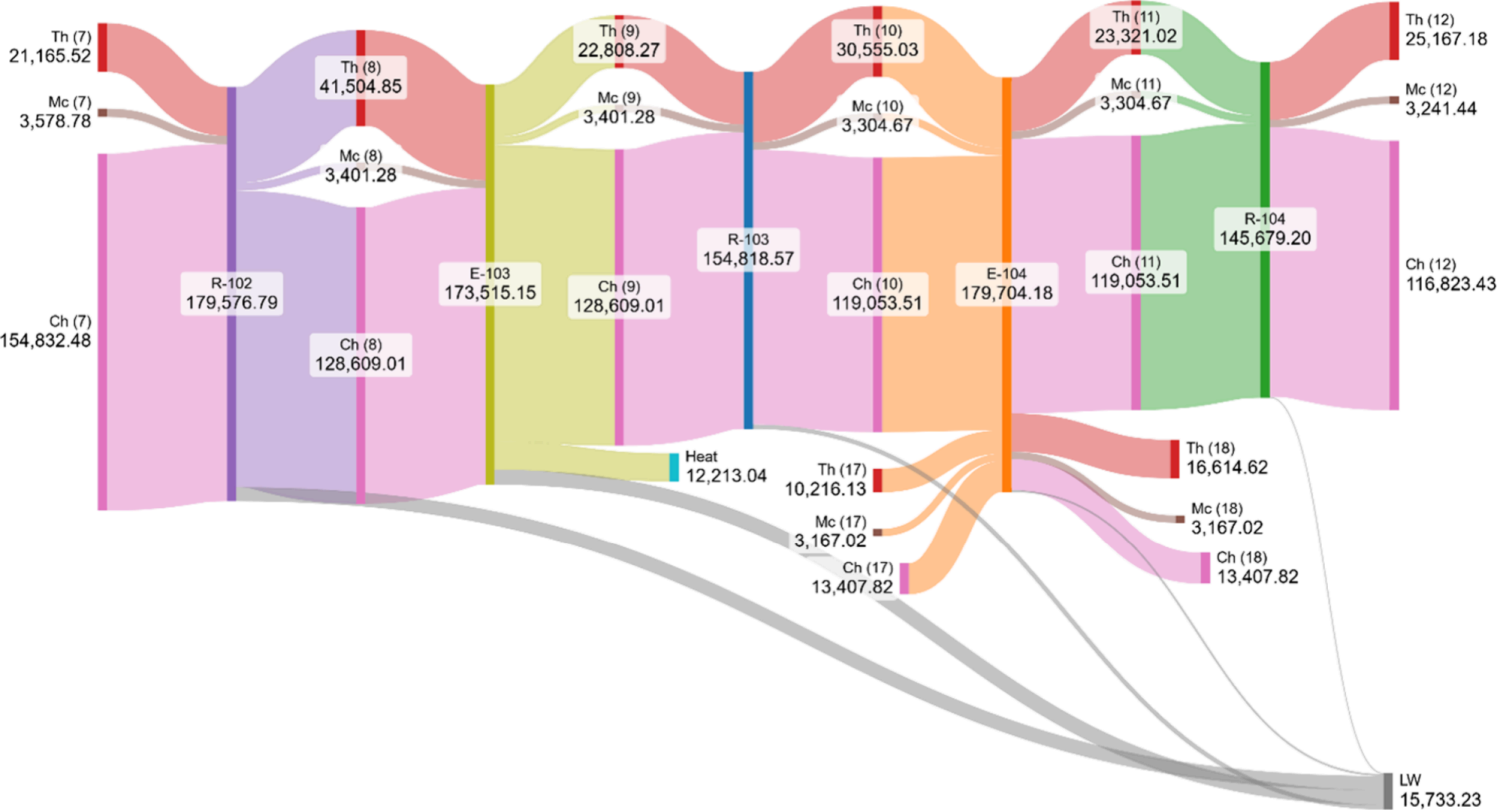
Exergy distribution and irreversibilities in R-102–R-104 and their intercoolers.

In R-102, a large inflow of chemical exergy (154,832 kW) is partially consumed as the oxidation of SO_2_ to SO_3_ proceeds, releasing a significant amount of thermal exergy (41,505 kW). This exothermic step is accompanied by irreversibilities, with lost work of approximately 6,062 kW. The hot effluent then passes through E-103, where the thermal exergy decreases substantially (from 41,505 kW to 22,808 kW) as heat is recovered, of which 12,213 kW is usefully employed for steam generation. However, the exchanger also accounts for relevant exergy destruction, with around 6,484 kW lost due to finite temperature differences in heat transfer. In R-103, the stream undergoes further chemical exergy reduction (128,609 kW to 119,054 kW), accompanied by the release of 30,555 kW of thermal exergy and irreversibilities of about 1,905 kW. E-104 then removes part of this heat, reducing the thermal exergy from 23,321 kW to 16,615 kW, with useful recovery but also 836 kW of lost work. Finally, R-104 continues the reaction, with chemical exergy further reduced to 116,823 kW, generating 25,167 kW of thermal exergy and incurring a modest 447 kW of irreversibilities.

Overall, this section of the plant reveals a stepwise cascade of chemical exergy conversion into thermal exergy, with heat exchangers enabling energy recovery but also introducing notable destruction. Compared to the sulfur burner (R-101), where irreversibilities are dominated by the chemical reaction constraint, the catalytic reactors and their intercoolers exhibit a more balanced interplay between chemical consumption, heat recovery, and thermal exergy destruction, emphasizing the importance of catalyst efficiency and heat integration in minimizing losses.

Figure 12 presents the Grassmann diagram for Mix-3, E-107, and the first absorber tower (T-102). The figure highlights the large chemical exergy stream entering Mix-3 (1,109,336 kW), which is only slightly modified before passing to E-107 and T-102. In E-107, part of the thermal exergy (5,553 kW) is reduced, with useful heat recovery (4,007 kW) but also noticeable irreversibilities (17,521 kW lost work). The tower T-102 then processes a significant share of chemical exergy (1,185,933 kW), while also being responsible for additional losses (5,278 kW). Overall, this section of the plant exhibits modest conversion of chemical into thermal exergy but contributes substantially to global irreversibilities, with a total of nearly 29,608 kW of lost work, underlining the role of heat recovery and absorption processes as secondary, yet important, inefficiency hotspots compared to the main reactors.

**Figure 12:**
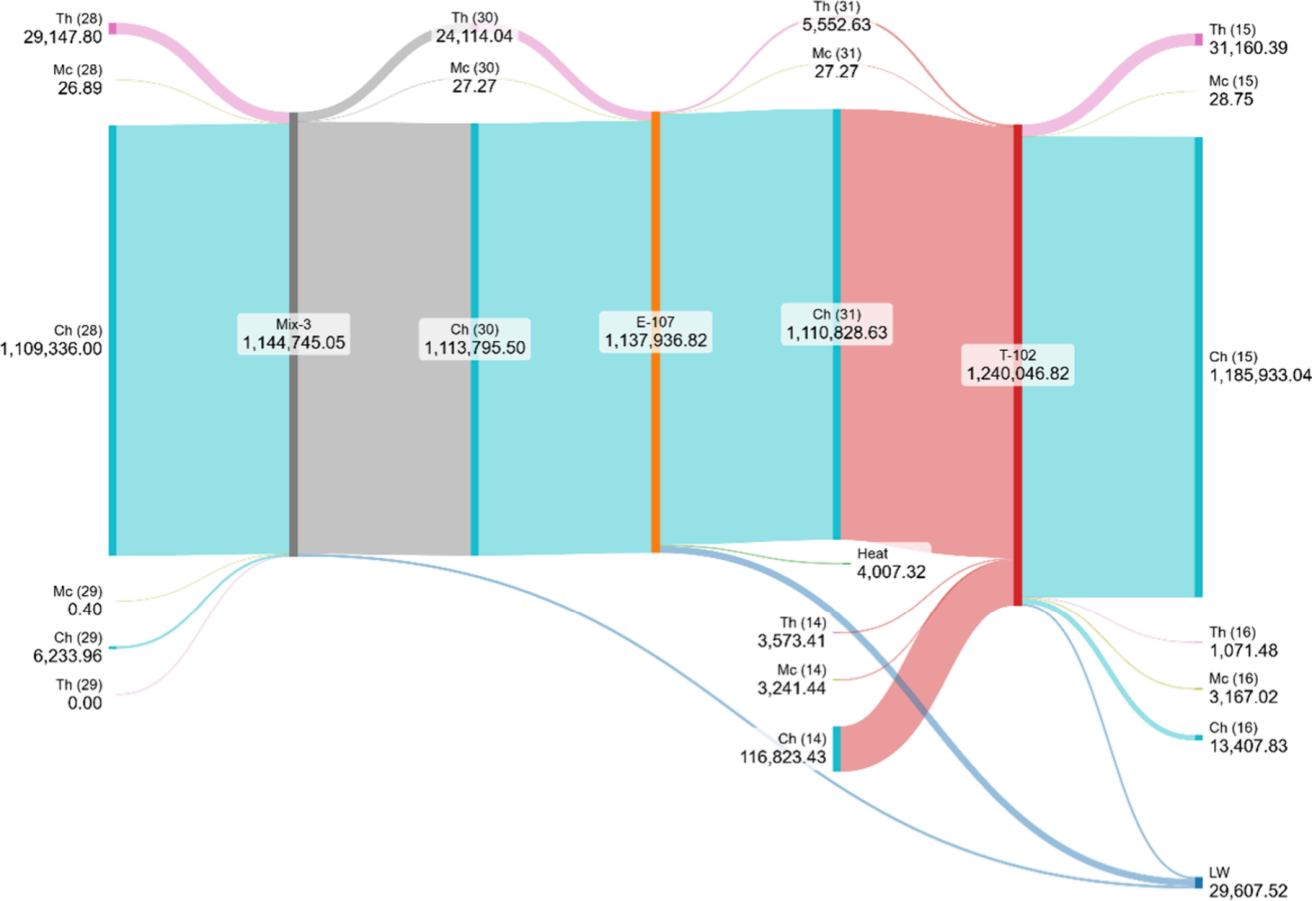
Exergy distribution and lost work in the absorption section (Mix-3, E-107, and T-102).

Thinking of strategies to reduce lost work, one option that can be implemented without altering the process-side operating conditions is to increase the pressure of the steam generated from the heat recovered in the reaction systems. In industrial sulfuric acid plants, steam is typically produced at pressures between 40 and 60 bar. In the base case, high-pressure steam was generated at 42 bar. If the pressure were increased to 60 bar in heat exchangers E-102, E-103, and E-108, the total lost work would decrease by 2,381 kW, corresponding to a 1.33% reduction relative to the initial value. When steam expansion for power generation is considered, the benefit increases to a 16% gain in power output.

Another measure was tested by focusing on heat exchangers E-106 and E-107, using the UniSim Design Optimizer to determine their optimal outlet temperatures. For E-106, the outlet temperature was allowed to vary between 160 °C and 200 °C, while for E-107 it was set to vary between 50 °C and 100 °C, with the objective of minimizing the total lost work of the plant and constraining lost work to be positive. The resulting optimal outlet temperatures were 170 °C for E-106 and 77.5 °C for E-107. However, the reduction in lost work was marginal, amounting to only 963.4 kW, or 0.54% of the initial total lost work.

An additional attempt focused on the furnace (R-101) and heat recovery exchanger (E-102) section of the plant, which is responsible for the largest share of lost work. To reduce the temperature difference between the hot gas stream generated in the furnace (by oxidizing sulfur to SO_2_) and the heat recovery system, the sulfur feed was split into three equimolar flow rates and directed to three furnaces operating in series with respect to the air stream. Two heat exchangers were placed between these furnaces to recover the released heat and generate high-pressure steam (42 bar), as illustrated in Figure 13.

**Figure 13:**
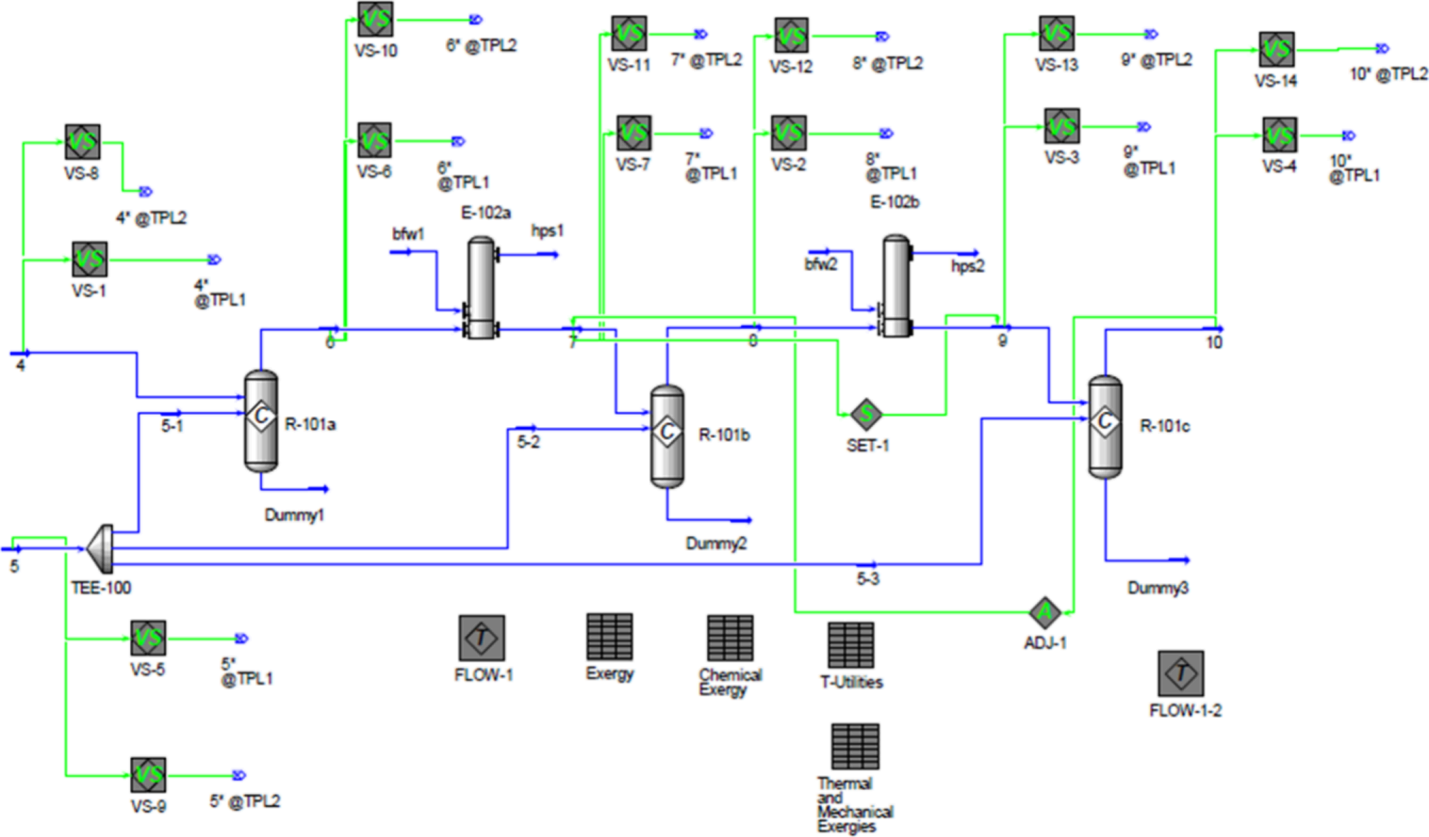
Schematic representation of the composite furnace-heat recovery system with three furnaces in series and intermediate heat exchangers (E-102a and E-102b).

The outlet temperatures of streams 7 and 9 were constrained to be equal and were adjusted so that the outlet temperature of the last furnace in the composite system matched the outlet temperature of the furnace in the base case. In each furnace, the sulfur feed was fully converted to SO_2_. The outlet temperatures of furnaces R-101a, R-101b, and R-101c were determined to be 418 °C, 420.4 °C, and 423 °C, respectively, while the temperatures of streams 7 and 9 were fixed at 150.7 °C. Figure 14 presents the Grassmann diagram of this composite configuration. When comparing the total lost work, it is observed that the sum of the lost work of R-101 and E-102 in the base case equals the combined lost work of R-101a, R-101b, R-101c, and E-102a/E-102b in the modified system. The same observation holds for the heat duty of E-102 when compared to the combined heat exchanged by E-102a and E-102b. Even if the number of furnaces in series were further increased, the results would remain unchanged. This supports the conclusion that the combined lost work of R-101 and E-102 is unavoidable, since further process rearrangements within reasonable bounds does not lead to improvements. It is also endogenous, as the combined lost work of R-101 and E-102 remains constant regardless of whether the furnace is split or additional heat exchangers are introduced. This demonstrates that the destruction is inherent to the furnace/heat recovery section itself, rather than being imposed by upstream or downstream units.

**Figure 14:**
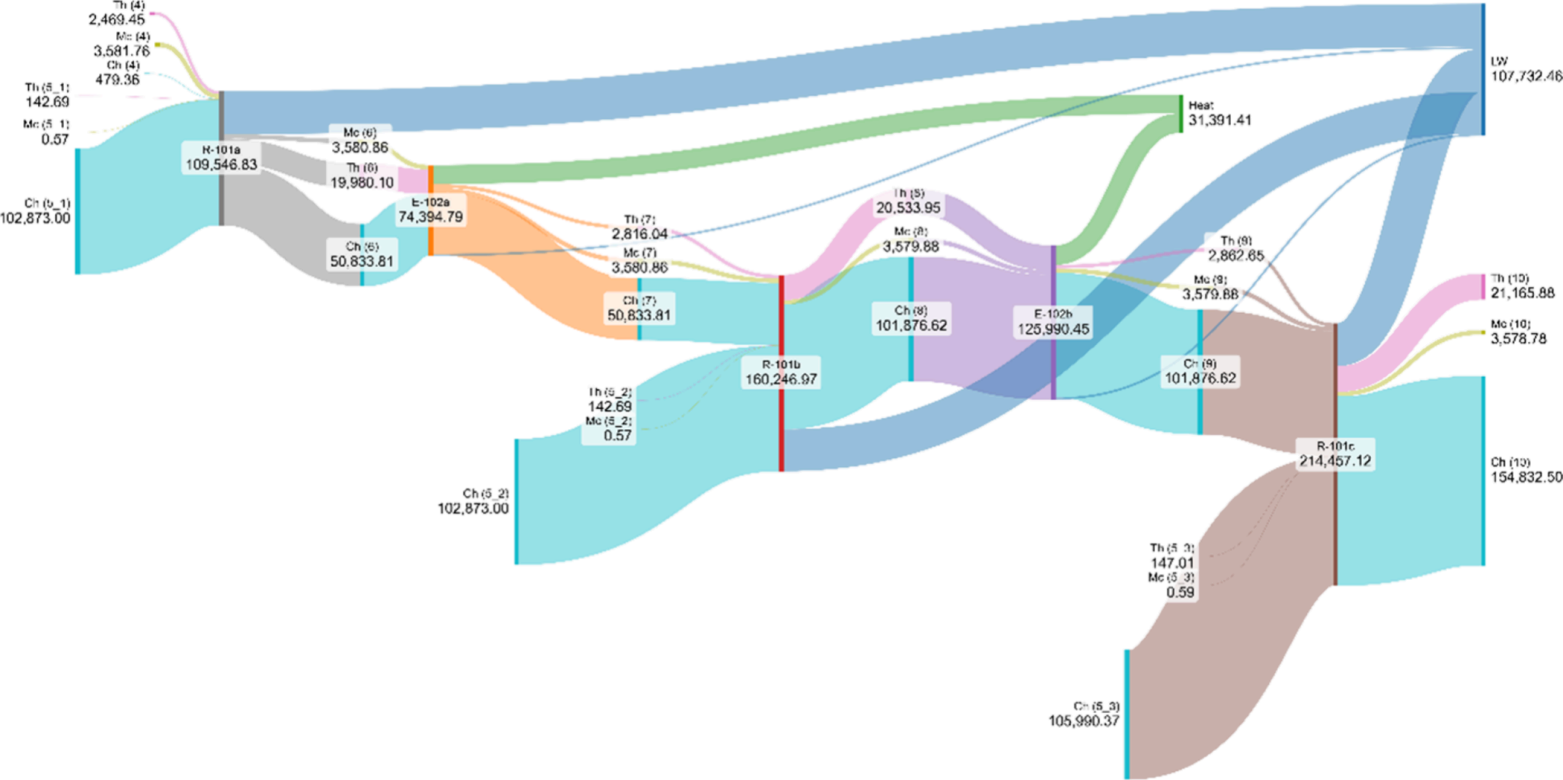
Grassmann diagram of the composite furnace-heat recovery system illustrating exergy flows and lost work distribution.

## 6 Conclusions and Future Developments

This work presented a detailed exergy analysis of sulfuric acid production through the double contact process, carried out using UniSim Design as a simulation platform. By adopting a didactic and systematic approach, the study demonstrated how fundamental exergy concepts can be effectively applied to a realistic industrial case, bridging the gap between thermodynamic theory and practical process simulation. The workflow proved robust and transparent, as UniSim enabled consistent calculation of enthalpies, entropies, and exergies directly within the simulator, consolidating all steps into a single environment.

The results confirmed the dominant role of the sulfur-burning furnace (R-101) as the major source of lost work, reflecting the strongly irreversible nature of the initial sulfur oxidation reaction. Alongside R-101, heat exchangers such as E-102 and E-107 were identified as significant contributors to overall exergy destruction, largely due to finite temperature differences during heat transfer. Together, these three units accounted for more than 70% of the total plant irreversibilities, emphasizing their importance as primary targets for efficiency improvement.

Three improvement strategies were tested. First, increasing the pressure of the steam generated in E-102, E-103, and E-108 from 42 to 60 bar reduced the total lost work by 2,381 kW (1.33%). More importantly, when considering steam expansion for power generation, this change yielded a much greater benefit, with a 16% increase in power output. Second, optimization of outlet temperatures in E-106 and E-107, using the UniSim Optimizer, resulted in only marginal gains, with lost work reduced by 963.4 kW (0.54%). Third, the furnace/heat recovery section was reconfigured by dividing the sulfur feed into three streams and routing them through three furnaces arranged in series with interstage heat exchangers. The analysis showed that the combined lost work of R-101 and E-102 in this configuration was equal to that of the base case. Even when increasing the number of furnaces, results remained unchanged, indicating that the irreversibilities in this section are both unavoidable and endogenous, being inherent to the reaction and heat recovery system rather than imposed by upstream or downstream units.

At the plant level, the overall exergy efficiency was relatively low (21.02%) because the exergy of the outlet gas stream was excluded, as their work potential is not recovered. Including them would increase the efficiency to 48.25%. This highlights the importance of clearly defining system boundaries when evaluating process performance.

Looking ahead, future work should expand the scope by incorporating exergoeconomic and exergoenvironmental analyses, enabling a combined thermodynamic, economic, and environmental assessment of the process. Applying advanced exergy decomposition into avoidable/unavoidable and endogenous/exogenous parts will provide sharper insights into feasible improvements. Finally, coupling the methodology with dynamic simulation or digital twin frameworks would enhance its application in real-time monitoring, predictive maintenance, and advanced process control, aligning the approach with emerging industrial practices.

In summary, the study not only identified the main sources of exergy destruction in the sulfuric acid process but also tested practical strategies for improvement, confirming both the potential and the limitations of efficiency enhancement. By focusing on fundamental principles while demonstrating applied simulations, this work strengthens the educational and practical dissemination of exergy analysis and sets the stage for future extensions toward advanced, optimization-driven, and sustainability-oriented process evaluation.

## Supplementary Material



## Notes

Additional details can be found in the supporting material on the UniSim Design model configuration; reaction kinetics and reactor setup for the catalytic beds; complete stream tables including temperatures, pressures, compositions, enthalpies, and entropies (Tables S1–S3); calculated physical, chemical, and total exergies of all process streams (Table S4); Grassmann diagrams for major process units (Figures S1–S4); supplementary calculation for chemical exergy of vapor, liquid, and vapor–liquid streams (PDF)
