# Supplementary material for: Exergy Analysis of Sulfuric Acid Production: A Systematic Framework Using UniSim Design
Source: ACS Omega. 2026 Feb 27;11(9):15145–63. doi: 10.1021/acsomega.5c12233 (PMC12980436; doi:10.1021/acsomega.5c12233)
Supplement: Supplementary file 1 [file ao5c12233_si_001.pdf]

Exergy Analysis of Sulfuric Acid Production: A Systematic Framework Using UniSim Design

Ulysses Guilherme Ferreira[a], Thiago Vaz da Costa[a], Sérgio Mauro da Silva Neiro[a*]

[a] *School of Chemical Engineering, Uberlândia Federal University (UFU), Av João Naves de Ávila, 2121, bloco 5K, sala 225, Santa Mônica, 38408-100, Uberlândia-MG, Brazil*

*corresponding author: srgneiro@ufu.br

Supplementary Materials

The supplementary materials provide detailed datasets and complementary analyses that support the results and discussions presented in the main text. These additional resources include full tabulations of process stream properties, thermodynamic state functions, and calculated exergies for the sulfuric acid production flowsheet modeled in UniSim Design. Furthermore, Grassmann diagrams are presented for individual equipment and process sections, offering a visual representation of exergy flows and irreversibilities that enrich the quantitative evaluation discussed in the manuscript.

Supplementary Material S1 - Additional information on the converter

The section presents details regarding the kinetic modeling of the main converter, where sulfur dioxide is oxidized to sulfur trioxide. The reaction rate expression was adapted from Froment, Bischoff, and De Wilde (2011) and reformulated in the Langmuir–Hinshelwood form to fit the modeling framework of UniSim Design, as shown in Equation (S1). The reaction rate is expressed in $\mathrm{kmol/(m^3h)}$, with partial pressure specified in atm. The kinetic and adsorption constants used in the model are defined by Equations (S2) through (S5). Table S1 provides the main geometric and catalyst properties of each converter bed, including length, diameter, number

of tubes, wall thickness, void fraction, particle diameter, and catalyst density. These data are fundamental for reproducing the reactor's behavior in terms of reaction kinetics, pressure drop, and overall conversion performance.

$$r = \frac{k_1 p_{SO_2} p_{O_2} - k_2 p_{SO_3} p_{O_2}^{1/2}}{\left(1 + K_{SO_2} p_{SO_2} + K_{SO_3} p_{SO_3}\right)^2} \tag{S1}$$

where the kinetic constants k_1 and k_2, along with the adsorption constants K_{SO_2} and K_{SO_3}, are defined by Eqs. (S2)-(S5).

$$k_1 = 8{,}160{,}484 e^{-\frac{45{,}503(\mathrm{kJ/kmol})}{RT}} \tag{S2}$$

$$k_2 = 3.55 \times 10^{11} e^{-\frac{1.3945 \times 10^5(\mathrm{kJ/kmol})}{RT}} \tag{S3}$$

$$K_{SO_2} = 4.76 \times 10^{-5} e^{\frac{71{,}658.4(\mathrm{kJ/kmol})}{RT}} \tag{S4}$$

$$K_{SO_3} = 6.94 \times 10^{-32} e^{\frac{437{,}283(\mathrm{kJ/kmol})}{RT}} \tag{S5}$$

Froment, G. F.; Bischoff, K. B.; De Wilde, J., Chemical reactor analysis and design, 3rd edition, 2011, 860 pages, Wiley

Table S1 provides the dimensions and catalyst-related details for each catalyst bed.

Catalyst Bed	Length (m)	Diameter (m)	# of tubes	Wall Thickness (m)	Void Fraction	Particle Diameter (m)	Catalyst Density ($\mathrm{kg/m^3}$
1	0.78	16.25	1	0.002	0.48	0.01	884.0
2	0.90	16.25	1	0.002	0.48	0.01	884.0
3	1.07	16.25	1	0.002	0.48	0.01	884.0
4	1.34	16.25	1	0.002	0.48	0.01	884.0

Supplementary Material S2 – Sulfuric Acid Process Stream Summary

Table S2 summarizes the thermodynamic and compositional characteristics of all process streams represented in the flowsheet of Figure 6 in the main paper. It lists each stream's vapor fraction, temperature, pressure, total molar flow, and individual component molar flows. These comprehensive data describe the state of the process at every stage and constitute the basis for calculating energy and exergy balances. The information ensures full reproducibility of the material balance and allows independent verification of the simulation results.

Table S2 - Summary of process streams corresponding to the flowsheet illustrated in Figure 6.

Stream	1	2	3	4	5
Vapor fraction	1.000	1.000	1.000	1.000	0.000
Temperature (°C)	25.0	63.2	45.0	143.0	132.0
Pressure (kPa)	101.3	140.0	140.0	140.0	140.0
Molar flow (kmol/h)	16089.4	16089.4	16092.3	16092.3	1874.0
Comp molar flow (kmol/h)					
H_2O	205.9443	205.9443	208.7539	208.7539	0.0000
H_2SO_4	0.0000	0.0000	0.0000	0.0000	0.0000
S	0.0000	0.0000	0.0000	0.0000	1874.0097
N_2	12556.1647	12556.1647	12556.1796	12556.1796	0.0000
SO_3	0.0000	0.0000	0.0086	0.0086	0.0000
O_2	3327.2871	3327.2871	3327.3110	3327.3110	0.0000
SO_2	0.0000	0.0000	0.0076	0.0076	0.0000

Table S2 - Summary of process streams corresponding to the flowsheet illustrated in Figure 6 - *continued*.

Stream	6	7	8	9	10
Vapor fraction	1.000	1.000	1.000	1.000	1.000
Temperature (°C)	909.3	423.0	630.4	440.0	521.9
Pressure (kPa)	140.0	140.0	139.4	139.4	138.8
Molar flow (kmol/h)	16092.3	16092.3	15500.3	15500.3	15267.1
Comp molar flow (kmol/h)					
H_2O	208.7539	208.7539	208.7539	208.7539	208.7539
H_2SO_4	0.0000	0.0000	0.0000	0.0000	0.0000
S	0.0000	0.0000	0.0000	0.0000	0.0000
N_2	12556.1796	12556.1796	12556.1796	12556.1796	12556.1796
SO_3	0.0086	0.0086	1183.8796	1183.8796	1650.3540
O_2	1453.3013	1453.3013	861.3683	861.3683	628.1314
SO_2	1874.0173	1874.0173	690.1521	690.1521	223.6786

Table S2 - Summary of process streams corresponding to the flowsheet illustrated in Figure 6 - *continued*.

Stream	11	12	13	14	15
Vapor fraction	1.000	1.000	1.000	1.000	0.000
Temperature (℃)	445.0	465.1	309.1	166.0	116.8
Pressure (kPa)	138.8	138.1	138.1	138.1	137.8
Molar flow (kmol/h)	15267.1	15210.2	15210.2	15210.2	26517.2
Comp molar flow (kmol/h)					
H_2O	208.7539	208.7539	208.7539	208.7539	1.3457
H_2SO_4	0.0000	0.0000	0.0000	0.0000	26514.2144
S	0.0000	0.0000	0.0000	0.0000	0.0000
N_2	12556.1796	12556.1796	12556.1796	12556.1796	0.0608
SO_3	1650.3540	1764.0859	1764.0859	1764.0859	1.1251
O_2	628.1314	571.2655	571.2655	571.2655	0.0223
SO_2	223.6786	109.9468	109.9468	109.9468	0.3886

Table S2 - Summary of process streams corresponding to the flowsheet illustrated in Figure 6 - *continued*.

Stream	16	17	18	19	20
Vapor fraction	1.000	1.000	1.000	1.000	1.000
Temperature (℃)	116.8	320.3	420.0	443.7	135.0
Pressure (kPa)	137.8	137.8	137.8	137.7	137.7
Molar flow (kmol/h)	13701.5	13701.5	13701.5	13648.9	13648.9
Comp molar flow (kmol/h)					
H_2O	412.6679	412.6678	412.6678	412.6678	412.6678
H_2SO_4	0.0000	0.0000	0.0000	0.0000	0.0000
S	0.0000	0.0000	0.0000	0.0000	0.0000
N_2	12556.1757	12556.1757	12556.1757	12556.1757	12556.1757
SO_3	51.5133	51.5133	51.5133	156.7412	156.7412
O_2	571.2640	571.2640	571.2640	518.6501	518.6501
SO_2	109.9217	109.9216	109.9216	4.6937	4.6937

Table S2 - Summary of process streams corresponding to the flowsheet illustrated in Figure 6 - *continued*.

Stream	21	22	23	24	25
Vapor fraction	1.000	0.000	0.000	0.000	0.000
Temperature (℃)	83.1	83.1	62.0	62.0	25.0
Pressure (kPa)	137.7	137.7	137.7	137.7	140.0
Molar flow (kmol/h)	14839.1	19650.0	45105.8	19491.6	1504.8
Comp molar flow (kmol/h)					
H_2O	1759.0631	4.4924	5.2765	2.2801	1504.7882
H_2SO_4	0.0000	19645.4260	45100.4371	19489.2453	0.0000
S	0.0000	0.0000	0.0000	0.0000	0.0000
N_2	12556.1686	0.0175	0.0241	0.0104	0.0000
SO_3	0.5519	0.0153	0.0153	0.0066	0.0000
O_2	518.6590	0.0082	0.0396	0.0171	0.0000
SO_2	4.6861	0.0134	0.0134	0.0058	0.0000

Table S2 - Summary of process streams corresponding to the flowsheet illustrated in Figure 6 - *continued*.

Stream	26	27	28	29	30
Vapor fraction	0.000	0.000	0.000	0.000	0.052
Temperature (°C)	61.5	82.0	116.8	25.0	109.1
Pressure (kPa)	137.7	137.7	137.8	140.0	137.8
Molar flow (kmol/h)	20996.4	20996.4	24804.5	1916.5	26721.0
Comp molar flow (kmol/h)					
H_2O	1507.0683	1507.0683	1.2588	1916.5009	1917.7596
H_2SO_4	19489.2453	19489.2453	24801.7145	0.0000	24801.7145
S	0.0000	0.0000	0.0000	0.0000	0.0000
N_2	0.0104	0.0104	0.0569	0.0000	0.0569
SO_3	0.0066	0.0066	1.0525	0.0000	1.0525
O_2	0.0171	0.0171	0.0209	0.0000	0.0209
SO_2	0.0058	0.0058	0.3635	0.0000	0.3635

Table S2 - Summary of process streams corresponding to the flowsheet illustrated in Figure 6 - *continued*.

Stream	31	32	33	34	35
Vapor fraction	0.000	0.000	0.000	0.000	0.000
Temperature (°C)	66.0	116.8	62.0	112.2	62.0
Pressure (kPa)	137.8	137.8	137.7	137.7	137.7
Molar flow (kmol/h)	26721.0	1712.7	156.1	1868.8	25458.2
Comp molar flow (kmol/h)					
H_2O	1917.7596	0.0869	0.0183	0.1052	2.9781
H_2SO_4	24801.7145	1712.5000	156.0547	1868.5547	25455.1373
S	0.0000	0.0000	0.0000	0.0000	0.0000
N_2	0.0569	0.0039	0.0001	0.0040	0.0136
SO_3	1.0525	0.0727	0.0001	0.0727	0.0086
O_2	0.0209	0.0014	0.0001	0.0016	0.0224
SO_2	0.3635	0.0251	0.0000	0.0251	0.0076

Table S2 - Summary of process streams corresponding to the flowsheet illustrated in Figure 6 - *continued*.

Stream	36	37	38
Vapor fraction	0.000	0.000	0.000
Temperature (°C)	45.0	45.9	45.9
Pressure (kPa)	137.7	140.0	137.7
Molar flow (kmol/h)	25458.7	25455.8	25455.8
Comp molar flow (kmol/h)			
H_2O	3.5937	0.7841	0.7841
H_2SO_4	25455.0112	25455.0112	25455.0112
S	0.0000	0.0000	0.0000
N_2	0.0216	0.0066	0.0066
SO_3	0.0086	0.0000	0.0000
O_2	0.0554	0.0315	0.0315
SO_2	0.0076	0.0000	0.0000

Supplementary Material S3 – Sulfuric Acid Process Streams Enthalpy and Entropy

Table S3 presents the molar enthalpy and entropy values for all process streams in the flowsheet. The enthalpy values, derived from UniSim Design, reflect the thermal condition of each stream, while the entropy values describe their degree of disorder and potential for energy degradation. Together, these quantities are used to compute the thermal and mechanical exergy components as detailed in the methodology section of the main text.

Table S3 – Enthalpy and entropy of process streams in Figure 6 flowsheet.

	Stream				
	1	2	3	4	5
$\bar{H}(T,P,z)$ kJ/kmol	-3102.49	-1983.34	-2559.92	348.95	-70453.11
$\bar{H}(T^0,P,z)$ kJ/kmol	-3102.49	-3105.26	-3147.29	-3147.29	-76211.91
$\bar{H}(T^0,P^0,z)$ kJ/kmol	-3102.49	-3102.49	-3144.52	-3144.52	-76214.96
$\bar{S}(T,P,z)$ kJ/kmol/K	152.54	153.38	151.76	159.73	37.72
$\bar{S}(T^0,P,z)$ kJ/kmol/K	152.54	149.84	149.85	149.85	21.19
$\bar{S}(T^0,P^0,z)$ kJ/kmol/K	152.54	152.54	152.55	152.55	21.19

	6	7	8	9	10
$\bar{H}(T,P,z)$ kJ/kmol	-7855.52	-24927.43	-25878.68	-32774.54	-33274.20
$\bar{H}(T^0,P,z)$ kJ/kmol	-37753.00	-37753.00	-46750.12	-46750.12	-50486.78
$\bar{H}(T^0,P^0,z)$ kJ/kmol	-37748.24	-37748.24	-46744.79	-46744.79	-50481.26
$\bar{S}(T,P,z)$ kJ/kmol/K	200.70	182.16	192.66	184.10	187.95
$\bar{S}(T^0,P,z)$ kJ/kmol/K	155.02	155.02	154.99	154.99	154.38
$\bar{S}(T^0,P^0,z)$ kJ/kmol/K	157.72	157.72	157.66	157.66	157.01

Stream	11	12	13	14	15
$\bar{H}(T,P,z)$ kJ/kmol	-36090.62	-36225.22	-41838.46	-46783.98	-927461.42
$\bar{H}(T^0,P,z)$ kJ/kmol	-50486.78	-51415.11	-51415.11	-51415.11	-960676.67
$\bar{H}(T^0,P^0,z)$ kJ/kmol	-50481.26	-51409.63	-51409.63	-51409.63	-960680.38
$\bar{S}(T,P,z)$ kJ/kmol/K	184.22	185.15	176.62	166.88	-104.78
$\bar{S}(T^0,P,z)$ kJ/kmol/K	154.38	154.18	154.18	154.18	-202.00
$\bar{S}(T^0,P^0,z)$ kJ/kmol/K	157.01	156.77	156.77	156.77	-202.00

Stream	16	17	18	19	20
$\bar{H}(T,P,z)$ kJ/kmol	-8431.29	-2199.96	938.27	941.89	-8679.02
$\bar{H}(T^0,P,z)$ kJ/kmol	-11471.53	-11471.53	-11471.53	-12281.62	-12281.62
$\bar{H}(T^0,P^0,z)$ kJ/kmol	-11164.94	-11164.94	-11164.94	-11970.56	-11970.56
$\bar{S}(T,P,z)$ kJ/kmol/K	157.62	170.46	175.35	176.38	158.87
$\bar{S}(T^0,P,z)$ kJ/kmol/K	148.37	148.37	148.37	148.19	148.19
$\bar{S}(T^0,P^0,z)$ kJ/kmol/K	152.19	152.19	152.19	152.03	152.03

Stream	21	22	23	24	25

$\overline{H}(T,P,z)$ kJ/kmol	-27061.98	-939411.98	-947086.06	-947086.06	-285246.93
$\overline{H}(T^0,P,z)$ kJ/kmol	-33019.56	-960589.44	-960665.24	-960665.24	-285246.93
$\overline{H}(T^0,P^0,z)$ kJ/kmol	-32681.14	-960593.14	-960668.95	-960668.95	-285247.48
$\overline{S}(T,P,z)$ kJ/kmol/K	158.00	-137.05	-159.06	-159.06	22.11
$\overline{S}(T^0,P,z)$ kJ/kmol/K	139.32	-201.98	-202.01	-202.01	22.11
$\overline{S}(T^0,P^0,z)$ kJ/kmol/K	142.80	-201.98	-202.00	-202.00	22.11

Stream	26	27	28	29	30
$\overline{H}(T,P,z)$ kJ/kmol	-899652.69	-892656.85	-927461.42	-285246.93	-881400.05
$\overline{H}(T^0,P,z)$ kJ/kmol	-912258.68	-912258.68	-960676.67	-285246.93	-912232.55
$\overline{H}(T^0,P^0,z)$ kJ/kmol	-912262.16	-912262.16	-960680.38	-285247.48	-912236.03
$\overline{S}(T,P,z)$ kJ/kmol/K	-145.62	-125.13	-104.78	22.11	-93.41
$\overline{S}(T^0,P,z)$ kJ/kmol/K	-185.94	-185.94	-202.00	22.11	-185.92
$\overline{S}(T^0,P^0,z)$ kJ/kmol/K	-185.94	-185.94	-202.00	22.11	-185.92

	Stream				
	31	32	33	34	35
$\overline{H}(T,P,z)$ kJ/kmol	-898080.27	-927461.42	-947086.06	-929100.41	-947086.06
$\overline{H}(T^0,P,z)$ kJ/kmol	-912232.55	-960676.67	-960665.24	-960675.55	-960665.24
$\overline{H}(T^0,P^0,z)$ kJ/kmol	-912236.03	-960680.38	-960668.95	-960679.26	-960668.95
$\overline{S}(T,P,z)$ kJ/kmol/K	-140.97	-104.78	-159.06	-109.01	-159.06
$\overline{S}(T^0,P,z)$ kJ/kmol/K	-185.92	-202.00	-202.01	-202.00	-202.01
$\overline{S}(T^0,P^0,z)$ kJ/kmol/K	-185.92	-202.00	-202.00	-202.00	-202.00

Stream	36	37	38
$\overline{H}(T,P,z)$ kJ/kmol	-953267.14	-953009.87	-953009.87
$\overline{H}(T^0,P,z)$ kJ/kmol	-960647.37	-960724.76	-960725.00
$\overline{H}(T^0,P^0,z)$ kJ/kmol	-960651.08	-960728.70	-960728.70
$\overline{S}(T,P,z)$ kJ/kmol/K	-178.04	-177.02	-177.02
$\overline{S}(T^0,P,z)$ kJ/kmol/K	-202.00	-202.03	-202.03
$\overline{S}(T^0,P^0,z)$ kJ/kmol/K	-202.00	-202.03	-202.03

Supplementary Material S4 – Sulfuric Acid Process Streams Physical and Chemical Exergies

Table S4 provides the calculated physical, chemical, and total exergies for all 38 process streams. The physical exergy reflects the deviation of each stream from environmental reference conditions, the chemical exergy accounts for the energy potential associated with its chemical composition, and the total exergy corresponds to their sum. These results form the quantitative foundation for the evaluation of exergy losses, irreversibilities, and equipment efficiencies presented in the paper.

Table S4 – Physical, chemical and total exergy of the process streams associated with the flowsheet illustrated in Figure 6.

	Stream				
	1	2	3	4	5
$\bar{B}^{Th}(T,P,z)$ kJ/kmol	0.00	66.27	18.87	552.44	830.58
$\bar{B}^{Mc}(T^0,P,z)$ kJ/kmol	0.00	801.27	801.27	801.27	3.29
$\bar{B}^{Ph}(T^0,P^0,z)$ kJ/kmol	0.00	867.54	820.15	1353.71	833.87
$\bar{B}^{Ch}(T,P,z)$ kJ/kmol	106.87	106.87	107.24	107.24	598850.00
$\bar{B}^{tot}(T^0,P,z)$ kJ/kmol	106.87	974.41	927.38	1460.95	599683.87
	6	7	8	9	10
$\bar{B}^{Th}(T,P,z)$ kJ/kmol	16279.54	4734.94	9639.63	5297.29	7204.91
$\bar{B}^{Mc}(T^0,P,z)$ kJ/kmol	800.61	800.61	789.96	789.96	779.24
$\bar{B}^{Ph}(T^0,P^0,z)$ kJ/kmol	17080.15	5535.55	10429.58	6087.25	7984.15
$\bar{B}^{Ch}(T,P,z)$ kJ/kmol	34637.58	34637.58	29869.84	29869.84	28072.96
$\bar{B}^{tot}(T^0,P,z)$ kJ/kmol	51717.72	40173.12	40299.42	35957.08	36057.11
	11	12	13	14	15
$\bar{B}^{Th}(T,P,z)$ kJ/kmol	5499.12	5956.64	2888.16	845.76	4230.37
$\bar{B}^{Mc}(T^0,P,z)$ kJ/kmol	779.24	767.19	767.19	767.19	3.90
$\bar{B}^{Ph}(T^0,P^0,z)$ kJ/kmol	6278.37	6723.83	3655.35	1612.96	4234.27
$\bar{B}^{Ch}(T,P,z)$ kJ/kmol	28072.96	27650.09	27650.09	27650.09	161003.64
$\bar{B}^{tot}(T^0,P,z)$ kJ/kmol	34351.33	34373.92	31305.44	29263.05	165237.92
	16	17	18	19	20
$\bar{B}^{Th}(T,P,z)$ kJ/kmol	281.52	2684.23	4365.39	4821.63	420.45
$\bar{B}^{Mc}(T^0,P,z)$ kJ/kmol	832.11	832.11	832.11	831.40	831.40
$\bar{B}^{Ph}(T^0,P^0,z)$ kJ/kmol	1113.64	3516.34	5197.51	5653.04	1251.86
$\bar{B}^{Ch}(T,P,z)$ kJ/kmol	3522.83	3522.83	3522.83	2961.74	2961.74
$\bar{B}^{tot}(T^0,P,z)$ kJ/kmol	4636.46	7039.17	8720.33	8614.78	4213.60
	21	22	23	24	25
$\bar{B}^{Th}(T,P,z)$ kJ/kmol	387.48	1817.27	775.89	775.89	0.00

$\overline{B}^{Mc}(T^0,P,z)$ kJ/kmol	699.46	3.89	3.89	3.89	0.75
$\overline{B}^{Ph}(T^0,P^0,z)$ kJ/kmol	1086.93	1821.16	779.78	779.78	0.75
$\overline{B}^{Ch}(T,P,z)$ kJ/kmol	965.43	160970.38	160989.38	160989.38	11710.00
$\overline{B}^{tot}(T^0,P,z)$ kJ/kmol	2052.36	162791.54	161769.16	161769.16	11710.75

	26	27	28	29	30
$\overline{B}^{Th}(T,P,z)$ kJ/kmol	584.17	1469.58	4230.37	0.00	3248.78
$\overline{B}^{Mc}(T^0,P,z)$ kJ/kmol	3.66	3.66	3.90	0.75	3.67
$\overline{B}^{Ph}(T^0,P^0,z)$ kJ/kmol	587.83	1473.25	4234.27	0.75	3252.45
$\overline{B}^{Ch}(T,P,z)$ kJ/kmol	149653.28	149653.28	161003.64	11710.00	150056.83
$\overline{B}^{tot}(T^0,P,z)$ kJ/kmol	150241.11	151126.53	165237.92	11710.75	153309.28

	Stream				
	31	32	33	34	35
$\overline{B}^{Th}(T,P,z)$ kJ/kmol	748.08	4230.37	775.89	3851.66	775.89
$\overline{B}^{Mc}(T^0,P,z)$ kJ/kmol	3.67	3.90	3.89	3.89	3.89
$\overline{B}^{Ph}(T^0,P^0,z)$ kJ/kmol	751.75	4234.27	779.78	3855.55	779.78
$\overline{B}^{Ch}(T,P,z)$ kJ/kmol	149657.12	161003.64	160989.38	161002.44	160989.38
$\overline{B}^{tot}(T^0,P,z)$ kJ/kmol	150408.87	165237.92	161769.16	164857.98	161769.16

	36	37	38
$\overline{B}^{Th}(T,P,z)$ kJ/kmol	235.89	258.27	258.29
$\overline{B}^{Mc}(T^0,P,z)$ kJ/kmol	3.89	4.14	3.89
$\overline{B}^{Ph}(T^0,P^0,z)$ kJ/kmol	239.78	262.41	262.18
$\overline{B}^{Ch}(T,P,z)$ kJ/kmol	160984.93	161004.24	161004.24
$\overline{B}^{tot}(T^0,P,z)$ kJ/kmol	161224.71	161266.65	161266.42

Supplementary Material S5 – Grassmann Diagrams of Selected Process Units

This section presents the Grassmann diagrams corresponding to the principal process units of the sulfuric acid production plant depicted in Figure 6. The diagrams illustrate the exergy flows and their distribution within each unit, thereby complementing the quantitative results provided in the main text and supporting the thermodynamic assessment of the process.

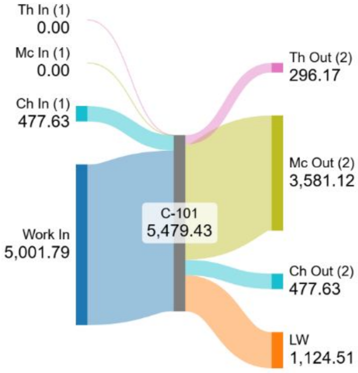

Figure S1 – Grassmann Diagram for compressor C-101.

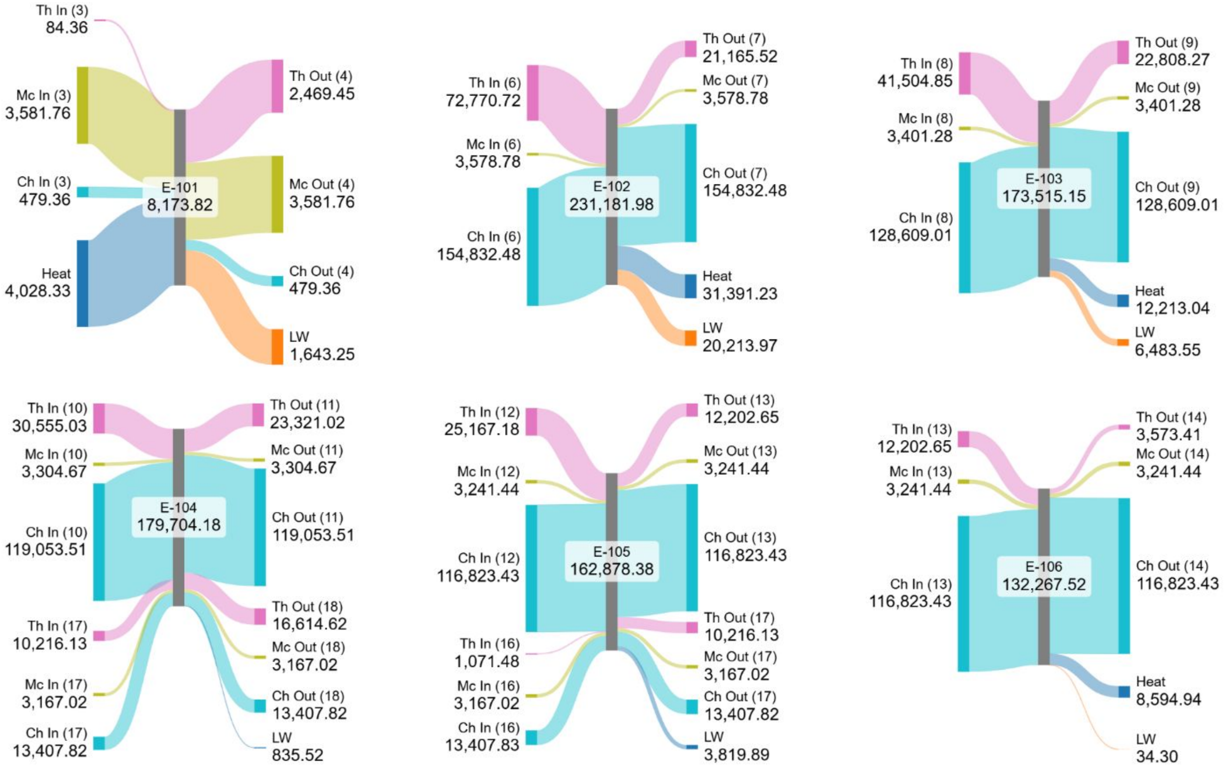

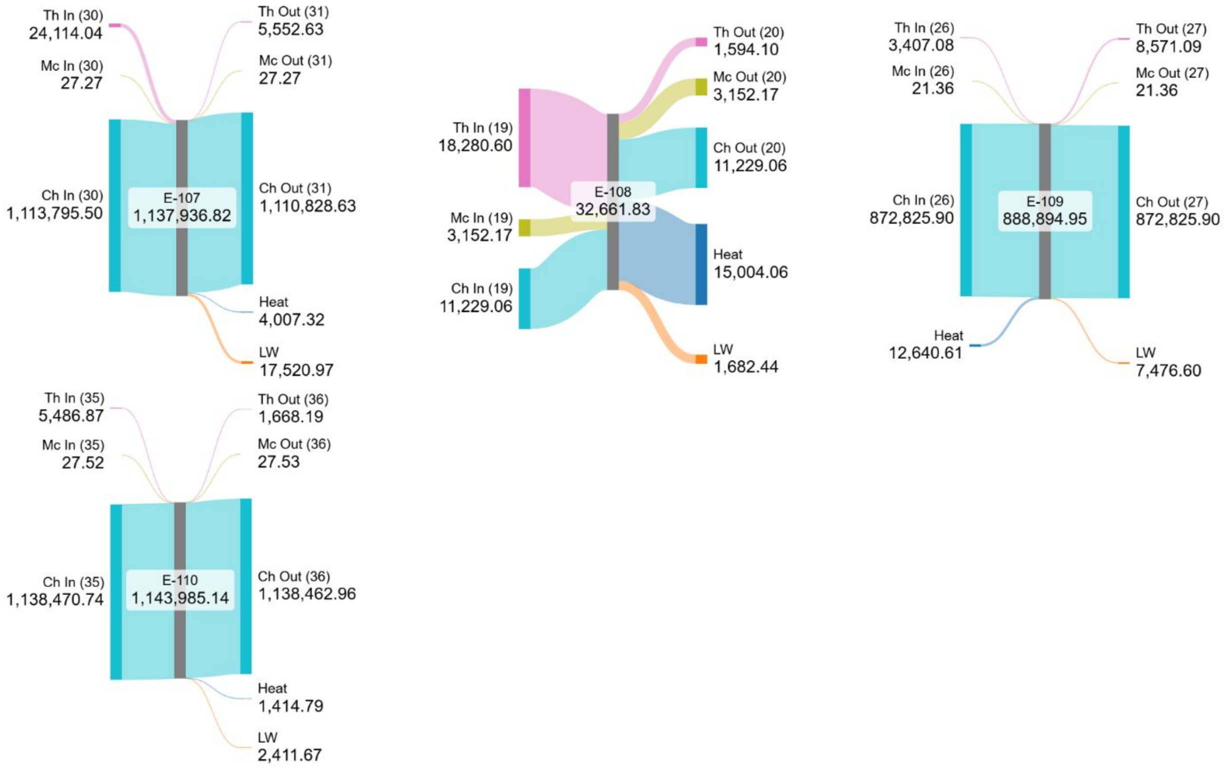

Figure S2 – Grassmann Diagram for heat exchangers.

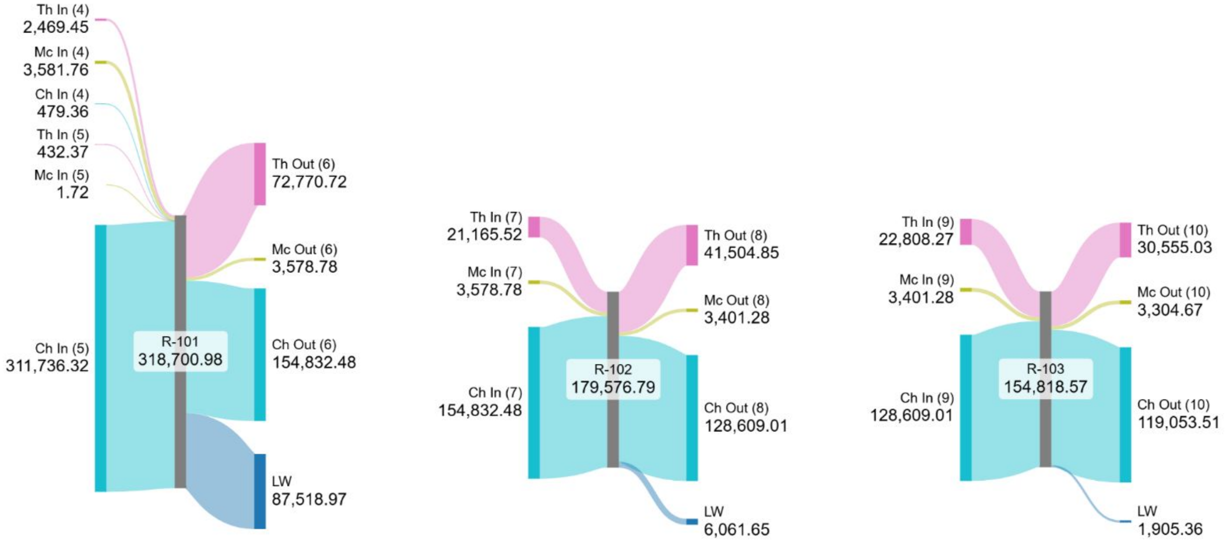

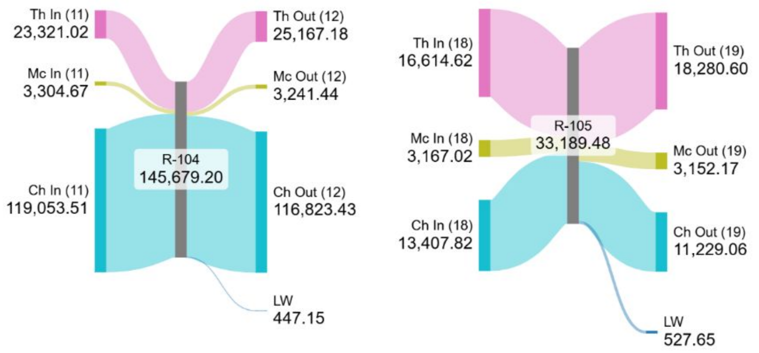

Figure S3 – Grassmann Diagram for reactors.

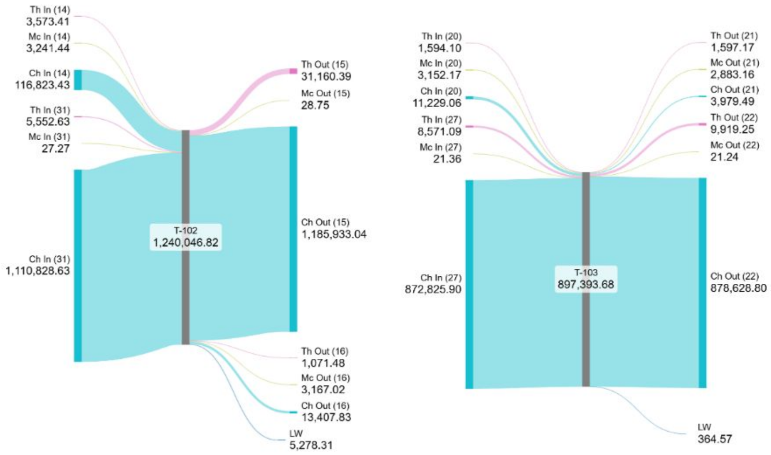

Figure S4 – Grassmann Diagram for absorption towers T-102 and T-103.
